# Effects of Tai Chi Chuan training on the QoL and psychological well-being in female patients with breast cancer: a systematic review of randomized controlled trials

**DOI:** 10.3389/fonc.2023.1143674

**Published:** 2023-05-01

**Authors:** Wenyuan Li, Fengming You, Qiaoling Wang, Yifeng Shen, Jundong Wang, Jing Guo

**Affiliations:** ^1^ Evidence Based Traditional Chinese Medicine Center of Sichuan Province, Hospital of Chengdu University of Traditional Chinese Medicine, Chengdu, China; ^2^ Traditional Chinese Medicine Regulating Metabolic Diseases Key Laboratory of Sichuan Province, Hospital of Chengdu University of Traditional Chinese Medicine, Chengdu, China; ^3^ Teaching and Research Office of Oncology in Traditional Chinese Medicine, Chengdu University of Traditional Chinese Medicine, Chengdu, China; ^4^ Departmental Office of Scientific Research, Hospital of Chengdu University of Traditional Chinese Medicine, Chengdu, China; ^5^ Clinical Medicine School, Chengdu University of Traditional Chinese Medicine, Chengdu, China

**Keywords:** Tai Chi Chuan, breast cancer, quality of life, psychological well-being, systematic review, meta-analysis

## Abstract

**Background:**

Tai Chi Chuan (TCC) may have a positive impact on physical and psychological well-being in breast cancer patients, but the evidence remains limited and inconclusive. This systematic review aims to evaluate the effects of TCC on the quality of life (QoL) and psychological symptoms in women patients with breast cancer.

**Methods:**

This review has been registered on PROSPERO (ID: CRD42019141977). Randomized controlled trials (RCTs) of TCC for breast cancer were searched from eight major English and Chinese databases. All trials included were analyzed in accordance with the Cochrane Handbook. The primary outcomes were QoL, anxiety, and depression in patients with breast cancer. Fatigue, sleep quality, cognitive function, and inflammatory cytokine were the secondary outcomes.

**Results:**

Fifteen RCTs involving a total of 1,156 breast cancer participants were included in this review. The methodological quality of included trials was generally poor. The pooled results suggested that TCC-based exercise could significantly improve QoL [standardized mean difference (SMD)=0.35, 95%CI: 0.15–0.55, *I* ^2 ^= 0, model: fixed, IV], anxiety [weighted mean difference (WMD)=−4.25, 95%CI: −5.88 to −2.63, *I* ^2 ^= 0, model: fixed, IV], and fatigue (SMD=−0.87, 95%CI: −1.50 to −0.24, *I* ^2 ^= 80.9%, model: random, DL) compared other controls, with moderate to low certainty of evidence. The improvement of QoL and fatigue by TCC was also clinically meaningful. However, TCC-based exercise failed to show any between-group differences in depression, sleep quality, cognitive function, and inflammatory cytokine. *Post-hoc* analysis revealed that TCC-based exercise outperformed the other exercise in improving shoulder function with very low certainty of evidence.

**Conclusion:**

Our findings manifested that TCC-based exercise is helpful for improving the QoL, anxiety, and fatigue in patients with breast cancer within the range of comparisons covered in this study. However, the results must be treated with great caution because of the methodological flaws of included trials. Larger, well-designed, and conducted randomized controlled trials with longer follow-up is warranted in the future to evaluate the important outcomes of TCC for breast cancer.

**Systematic review registration:**

https://www.crd.york.ac.uk/prospero/display_record.php?ID=CRD42019141977, identifier, CRD42019141977.

## 1 Introduction

Breast cancer is the most common cancer among women worldwide, contributing 24.5% of incidence and 15.5% of mortality by 2020 (1, 2). Moreover, the incidence and mortality of breast cancer are increasing at an alarming rate in some countries (3). Despite important advances in the understanding of subtypes and treatments, breast cancer remains a major health problem, including how to treat triple negative breast cancer and drug resistance (4, 5). The breast cancer experience can have considerable negative effects on women, both physically and psychologically (6). Long-term psychological distress, fatigue, cognitive impairment, sleep problems, and impaired quality of life (QoL) are common complaints among breast cancer patients (7–15). Impairments in these psychological areas collectively affect QoL through interrelated networks (cognition of body changes, self-shaping, emotions, etc.) (6, 16, 17). Therefore, it is undoubtedly of great significance to use QoL as an outcome for evaluating interventions. However, the construction of QoL is multi-dimensional and multi-disciplinary, and its conceptual scope and methodological characteristics are still very problematic (18). Although there are some more official or generic definitions, these definitions are obscure in explaining the conceptual scope of quality of life (19, 20). We reviewed the three most commonly used general quality of life scales (SF-36, EQ-5D, and WHOQOL-BREF) and a specific scale (FACT-B) for breast cancer; general health, physical function, pain, emotion and social/family well-being are common aspects of QoL that are more concerned (18, 21–24). These dimensions not only constitute quality of life but can also be interpreted as important factors affecting QoL.

Apart from genetic factor, aging, family history, reproductive factors, estrogen, and lifestyle are five important risk factors of breast cancer (25). However, the only factor we can directly modify is lifestyle. Physical activity (PA) has been increasingly recognized as an active lifestyle for preventing and improving breast cancer prognosis (26–28). It is also likely to be an effective adjunct to cancer therapy that can reduce the risk of both breast cancer-specific and all-cause mortality (29, 30). Other benefits of PA such as improving sequelae of breast cancer treatment, decreasing the recurrence, and improving survival have been shown in multiple studies (26, 30–32).

PA may improve physical and psychological factors during and after aggressive treatment for breast cancer (33, 34). A Cochrane review suggested that PA might have beneficial effects on the QoL, psychological well-being (anxiety and depression), sleep disorders, fatigue, and cognitive function of breast cancer survivors at different follow-up periods (35). The latest American Cancer Society guideline on nutrition and PA for cancer survivors suggested that people diagnosed with cancer should get PA assessment and counseling immediately to set appropriate exercise goals in order to cope with the agonizing treatment process that follows, with regard to PA’s benefit on clinical outcomes and patient-reported outcomes (36).

There are many ways in which PA may affect QoL, the most obvious being that PA improves physical function, on the basis that PA further relieves pain (37). Improvements in pain and physical function interact with self-perception and emotion in breast cancer patients (38, 39). Guided PA can also lead to social support and increased life satisfaction (40). Appropriate social support is important for cancer patients, especially breast cancer patients (41, 42). Participating in physical activity can also improve decision-making ability by improving cognition, and good decision-making plays an important role in the whole process of cancer (43). Transferable skills acquired during physical activity into health management may also be beneficial in improving cancer outcomes (44).

There are several evidence-based integrative therapies recommended by the Society for Integrative Oncology and endorsed by the American Society of Clinical Oncology to patients with breast cancer for improving QoL, performance or mental status, and psychological symptoms caused by anti-cancer treatment or cancer itself (45, 46). Integrative therapies, or the so-called complementary and alternative medicine, especially mind–body PA like TCC, were used stably by female breast cancer patients for the purpose of influencing well-being (47). Tai Chi Chuan (TCC) is a traditional Chinese exercise based on the philosophy of Yin and Yang, combining the essence of Chinese folk martial arts, breathing, meditation methods, and Traditional Chinese Medicine theories (48). As a potential and acceptable form of mind–body exercise, TCC has been widely practiced in both Eastern and Western countries and become the link between China and the world for cultural exchange since 1950s (49). Encouragingly, TCC has now gained global recognition, and the United Nations Educational, Scientific, and Cultural Organization Representative List of the Intangible Cultural Heritage of Humanity has inscribed TCC in 2020. Over the past decade, published clinical studies on TCC increased by 30%. Breast cancer is one of the most studied diseases of TCC exercise intervention. In the studies of TCC, psychological outcomes and QoL were commonly assessed (50).

Unfortunately, previous studies have not built a solid evidence base of whether TCC, as an adjuvant therapy, is beneficial to the physical and psychological well-being of breast cancer patients on QoL, depression, anxiety, fatigue, sleep quality, cognitive function, and other important outcomes (45). Several systematic reviews and meta-analyses have been done for the question (51–58). However, we found that it has been 2 years since the most recent review was published, and some new original studies were not included. Furthermore, the outcomes that we focused on and some methods applied in this study were different from those in previous reviews. It is necessary to produce an updated and rigorous systematic review to evaluate whether TCC-based exercise is superior to other exercise or non-exercise therapy on QoL and psychosomatic symptoms in women breast cancer patients, with a view to find new evidence.

## 2 Materials and methods

### 2.1 Study design

The study was registered on PROSPERO (ID=CRD42019141977), and the protocol has been published (59). We reported this review strictly following the Preferred Reporting Items for Systematic Reviews and Meta-Analysis (PRISMA) 2020 statement (60) see **Supplementary Table S1**.

### 2.2 Database and search strategy

We searched four English medical databases (Cochrane Library, PubMed, EMBASE, and Web of Science), four Chinese medical databases (China National Knowledge Infrastructure Database (CNKI), Sinomed, VIP Chinese Science and Technology Periodical Database, and Wan Fang Database), and psychological databases (APA PsycInfo and Psychology and Behavioral Sciences Colletion) systematically and comprehensively from their inceptions up to 30 September 2022. The development of search strategies followed the guidance of the Cochrane Handbook for Systematic Review of Interventions (61).

Search terms related to TCC and breast cancer. The following search terms were used including (“Tai Chi Chuan” OR “Tai Chi” OR “Tai Ji” OR “Tai Ji Quan” OR “Taijiquan” OR “Taiji” OR “Tai-ji”) AND (“breast cancer” OR “breast carcinoma” OR “breast neoplasm” OR “breast tumor”) AND “random∗.” To ensure a comprehensive search of the literature, we did manual retrieval of references of key trials and other systematic reviews published. The languages of the included trials were not limited. Detailed search strategies for each database are available in **Supplementary Table S2**.

### 2.3 Inclusion criteria

Studies should meet the following inclusion criteria (PICO format). (1) For the types of participants, all participants diagnosed as stage 0–III of primary breast cancer must be female over 18 years of age. The anti-cancer treatment received by participants could be any form of surgery, radiation therapy, chemotherapy, or hormone therapy. Additionally, participants should not be restricted to PA. (2) For the types of interventions, any types of TCC as major intervention were eligible, no matter the styles (such as Chen-, Yang-, Wu-, and Sun-style TCC) or forms (such as 24-, 8-, 18-, and 104-form). The duration of all interventions should be no less than 12 weeks, and the frequency of intervention should be at least once per week. (3) For the types of control, the controls could be any kind of exercise therapy or non-exercise therapy (such as standard support therapy, health education classes, cognitive behavioral therapy, psychosocial support, or usual care) other than TCC. The comparisons must ensure the comparability of TCC with other interventions, that is, when the experimental group uses TCC in combination with other interventions; the difference between the two groups can reflect the efficacy of TCC alone, rather than the combination of TCC and other interventions. In the three-arm trial, only the more conventional intervention with control-purpose was used. (4) For the types of outcomes, the primary outcomes were QoL and psychological symptoms (anxiety and depression), and the secondary outcomes included fatigue, sleep quality, cognitive function, and inflammatory cytokine. Shoulder function was also included as *post-hoc* analysis outcome for its important influence on psychological well-being. The measurements of these outcomes were not limited. (5) For the type of studies, the study design was strictly limited to RCTs.

### 2.4 Exclusion criteria

The following are the exclusion criteria: participants with other types of malignancy, literature duplicated and irrelevant, and reports without available data.

### 2.5 Data collection and extraction

The titles and abstracts of records searched were screened for eligibility after the duplicates were removed. Then, the full texts were obtained for final selection and data extraction.

We adopted a self-designed table for data extraction. Information extracted was as follows: (1) general information—research ID (the first author, year of publication), title, publication status, country, report sources, and funding; (2) methodological information—setting, design type, random sequence generation, allocation concealment, blinding, loss to follow-up, selective reporting, and baseline comparability; (3) participant information—sample size, age, diagnostic criteria, inclusion and exclusion criteria, course of disease, and status of cancer; (4) intervention information—details of intervention and control, duration and frequency of intervention, intervention instructor, adverse events, and follow-up; and (5) outcomes.

In the extracted data, standard errors are converted to standard deviations.

### 2.6 Assessment of methodological quality

We used the Cochrane Collaboration’s tool for assessing risk of bias (RoB) embedded in Review Manager 5.4.1 software to assess risk of bias in randomized trials (62, 63). Low risk of bias, high risk of bias, or unclear risk of bias were used as codes for the evaluation of these domains: random sequence generation, allocation concealment, blinding of participants and personnel, blinding of outcome assessment, incomplete outcome data, selective reporting, and other bias. We made the figures of risk of bias graph and summary for presenting.

Two reviewers (Wenyuan Li, WL and Jing Guo, JG) conducted study selection, data extraction, and methodological quality assessment independently. Any disagreements were resolved through discussions with another team member (Fengming You, FY). The Cochrane Handbook for Systematic Review of Interventions was consulted for all of these processes (61).

### 2.7 Data synthesis and analysis

Quantitative synthesis would be performed if the two authors (Qiaoling Wang, QW and Jundong Wang, JW) did not consider clinical heterogeneity in their discussion. In the absence of sufficient data to conduct meta-analyses, a narrative synthesis of the results was conducted. Else, we used the command **metan** embedded in Stata/SE 16.1 software for analyzing and synthesizing the outcomes (64). The data in full analysis set was preferentially used for pooling. Weighted mean difference (WMD)/standardized mean difference (SMD) and 95% confidence interval (CI) were calculated. WMD was used when trials measured the outcome on the same scale, while SMD was selected when trials measured the outcome on different scales. The SMDs was then re-expressed and presented as units of measures most relevant or used for breast cancer wherever possible if statistical significance was achieved, for the purpose of interpretating clinical significance. The last measurement before the end of each trial will be used for pooling.

Two-sided *p* ≤ 0.05 was considered as a criterion for statistical significance. *I* ^2^ > 50% was considered as an indication of substantial statistical heterogeneity. At this time, data would be analyzed using random-effect model with DerSimonian–Laird (DL) *tau^2^* estimator. Hartung–Knapp–Sidik–Jonkman variance correction to DerSimonian–Laird (HKSJ) *tau^2^* estimator and Biggerstaff–Tweedie (BT) approximate Gamma model were also used for sensitive analysis. Otherwise, a fixed-effect model would be used. The subscales of same measurement tool were also pooled by random-effect model with DL *tau^2^* estimator for exploring. Forest plots were used to show the synthesized results.

### 2.8 Subgroup analysis and sensitivity analysis

Subgroup analysis was deemed necessary. We performed subgroup analyses with different training duration and frequency of the TCC by random-effect model with DL *tau^2^* estimator. Besides comparing different methods of *tau^2^* estimation, we also performed sensitivity analyses by removing trials with high risk of bias, conducting influence meta-analysis (removing one trial from meta-analysis to detect the influence of the trial for effect), and comparing different measurement for the same outcome in one trial.

In order to explore the quantitative relationship between TCC practice duration and effect, we used the command **metareg** embedded in Stata/SE 16.1 software to perform *post-hoc* meta-regression analyses fitting SMD of QoL of different measure points with cumulative TCC practice time (weeks) (64). The model used restricted maximum likelihood iterative procedure to estimate the additive between studies variance *tau^2^*.

### 2.9 Publication bias

We would apply the Egger test for the for funnel plot asymmetry if there were meta-analysis including at least 10 trials (65).

### 2.10 Quality of evidence

This systematic review graded the evidence quality of clinical outcomes according to the Grades of Recommendations Assessment, Development and Evaluation (GRADE) approach (66). Five domains including risk of bias, indirectness of evidence, inconsistency of results, imprecision, and publication bias were considered for assessing evidence quality. We adopted the GRADE rubric developed by a research team from Australia to set the thresholds used to downgrade the certainty of the evidence and develop the summary of finding table see **Supplementary Table S3**.

## 3 Results

### 3.1 Selection of studies

**Figure 1:**
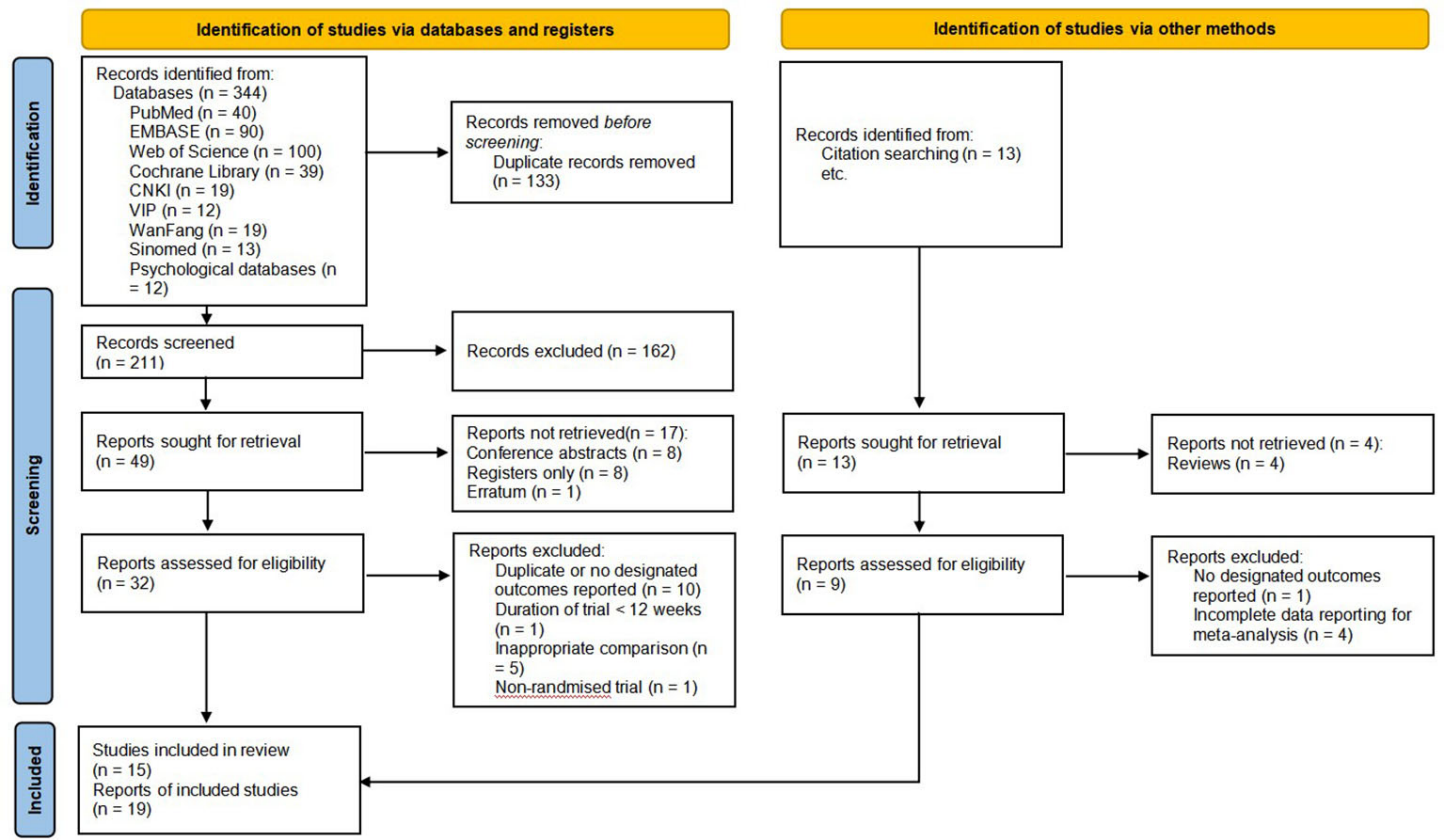
Flow diagram of identification, screening, and systematic review of Tai Chi Chuan training on the QoL and psychological well-being in women patients with breast cancer (60).

A total of 344 original records were identified from eight databases, of which 133 were excluded due to duplication and 162 were excluded due to irrelevance by reading titles and abstracts. When we sought the reports, there were 17 not retrieved (published as conference abstracts, just registration records, etc.). We assessed the remaining 32 reports, and 15 of them were included. After manually searching and screening the reference lists of the published RCTs and systematic reviews, four additional reports were retrieved and included, for a total of 19 (67–85). It should be emphasized that all included reports were from 15 RCTs, of which three reports were from the trial conducted by Mustian et al. (67–69), two reports were from the trial conducted by Larkey et al. (74, 75), and the other two reports were from the trial conducted by Zhu et al. (80, 81) We included different reports from the same RCTs because they had different outcomes. **Figure 1** shows the flow of studies through this review and reasons for exclusion.

A number of studies that might have been considered for eligibility in this review and studies that were included in previous systematic reviews but we did not ultimately include were also identified, and we gave reasons for not including them see **Supplementary Table S4**.

### 3.2 Characteristics of included trials

Of the 15 included RCTs, three originated from the USA (67–69, 74, 75, 82), 11 from China (70–73, 76, 77, 79–81, 83–85) and one from Thailand (78). Of all the 19 reports included, 16 were published in academic journals (67–79, 81, 82, 84), and the other three were dissertations (80, 83, 85). A total of 1,156 participants with American Joint Committee on Cancer (AJCC) stage of I–III breast cancer were included in this study, of whom 270 participants from five trials (67–69, 74, 75, 78, 82, 85) were survivors (the time since anti-cancer treatment completion ranged from 1 week to 5 years) and 886 participants from the other trials (70–73, 76, 77, 79–81, 83, 84) were undergoing chemotherapy shortly after surgery. The sample sizes of individual RCTs ranged from 21 to 149. All included RCTs were single-center trials. Baseline levels were generally comparable between the intervention and control groups for each trial, including sample size, participants’ age, cancer status, and current treatment status. In total, 582 participants were assigned to TCC-based exercises group and 574 to control group.

The intervention of included RCTs were different types of TCC (Yang-style TCC, Chen-style TCC, 24-form simplified TCC, 20-form TCC,18-form TCC, 8-form TCC, and Tai Chi Cloud Hands) alone (49–51, 55–57, 60, 64) or in combination with other exercise including routine rehabilitation training after surgery and strength training (52–54, 58, 59, 61–63, 65–67). The control of included RCTs were non-exercise therapy (including psychosocial therapy, cognitive behavioral therapy, and usual care) (67–69, 82, 84), sham Qigong without breathing and meditation (74, 75), routine rehabilitation training after surgery (70, 73, 76–79, 85), or combination of rehabilitation training after surgery and strength training or aerobics (71, 72, 80, 81, 83). All controls ensured the comparability between groups, allowing TCC to compare with psychotherapy, non-exercise, and aerobic or strength exercise under different intervention backgrounds.

The duration of TCC practice was from 12 to 24 weeks. The frequency of coaches supervised practice ranged from 3 to 14 sessions weekly, with 20–60 min per session. The weekly total practice time ranged from 90 to 300 min. The detailed characteristics of the eligible trials are shown in **Supplementary Table S5**.

### 3.3 Risk of bias in included studies

The methodological quality of the included studies was generally poor. All included trials mentioned “random,” 12 trials reported randomization sequence generation (67–71, 73–75, 77–84), seven of them used random number table (70, 71, 73, 77, 79–81, 83), three of them used computer generation (74, 75, 82, 84), and the other two trials used coin tossing (67–69) and random lottery (78); hence, they were evaluated as “low risk of bias.” The three trials that did not report randomization sequence generation were evaluated as “unclear risk of bias.” Six trials described the details associated with allocation concealment and were evaluated as “low risk of bias” (67–69, 73–75, 78, 82, 84). The other nine trials were evaluated as “unclear risk of bias”.

In terms of performance bias, only one trial using sham Qigong as a control has the possibility of blinding the participants and was evaluated as “low risk of bias” (74, 75). Other trials were evaluated as “high risk of bias.” Six of the included trials clearly reported the blinding of the outcome assessor and were evaluated as “low risk of bias” in terms of detection bias (71, 74, 75, 78, 80–83). One trial clearly reported that the outcome assessors were not blinded and were therefore rated as “high risk of bias” (67–69). The other trials were rated as “unclear risk of bias”.

**Figure 2:**
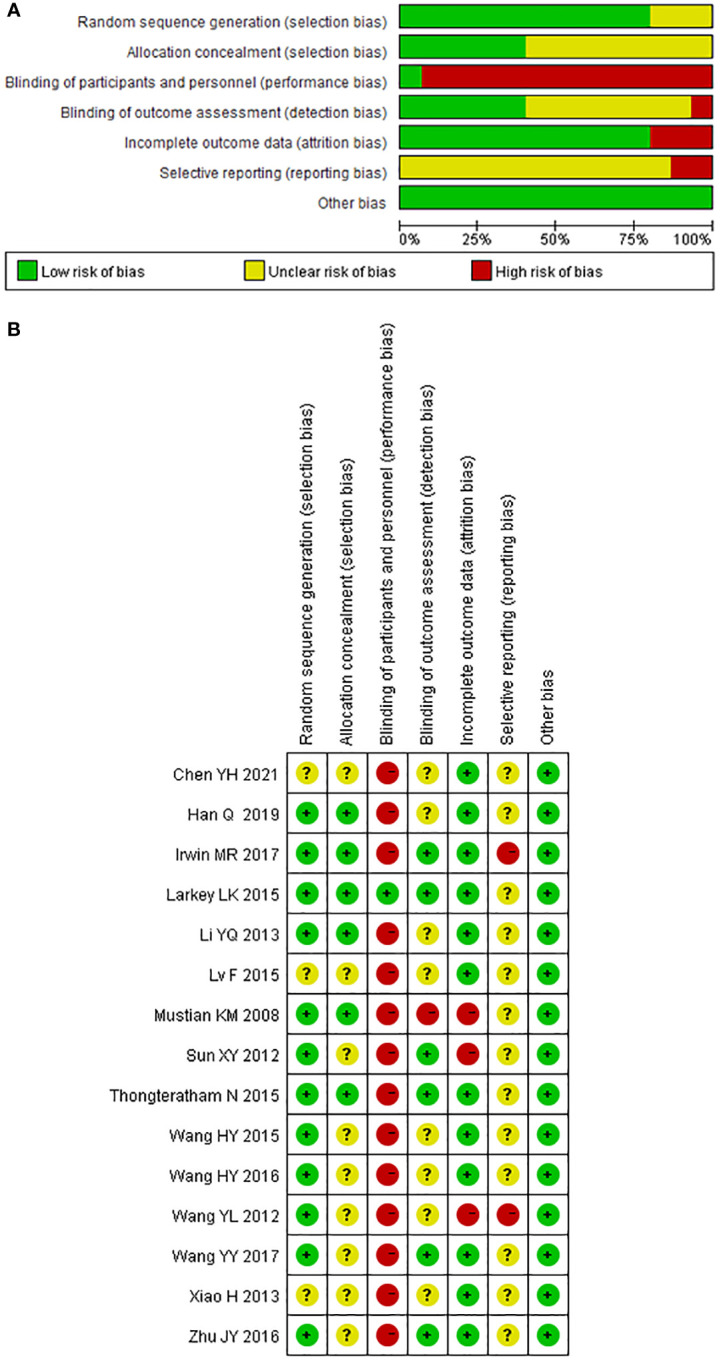
Risk of bias of included studies for the systematic review of Tai Chi Chuan training on the QoL and psychological well-being in female patients with breast cancer. **(A)** Risk of bias graph: the authors assessed each risk of bias item presented as percentages across all included studies. **(B)** Risk of bias summary: the authors judged each risk of bias item for each included study. +: low risk of bias; −: high risk of bias;?: unclear. The study ID consists of the first author’s surname, the capital initials of the first author’s first name, and the year the first report of the RCTs was published or submitted as dissertation.

Three trials that reported a large percentage of dropouts without performing an intent-to-treat (ITT) analysis were rated as “high risk of bias” in terms of attrition bias (67–71). Other trials were rated as “low risk of bias” due to the small dropout percentage and the relative balance between the two groups. All included trials did not report protocol registration and were rated as “unclear risk” of report bias except for the two trials that did not report all the outcomes mentioned in the methodology section and were therefore rated as “high risk of bias” (70, 82). We found no clues that might cause other bias. The methodological quality of the included trials is shown in **Figures 2A, B**.

### 3.4 Effects of interventions

#### 3.4.1 Primary outcomes

##### 3.4.1.1 QoL

A total of 10 trials reported QoL (67–76, 78, 79, 83), and the scales used to measure QoL included 36-Item Short Form Survey (SF-36) (67–69, 74–76), WHOQOL-BREF (70, 71, 73, 83), the Functional Assessment of Cancer therapy—Breast (FACT-B) (72, 78, 79), and the Functional Assessment of Chronic Illness Therapy—Fatigue (FACIT-F) (67–69).

**Figure 3:**
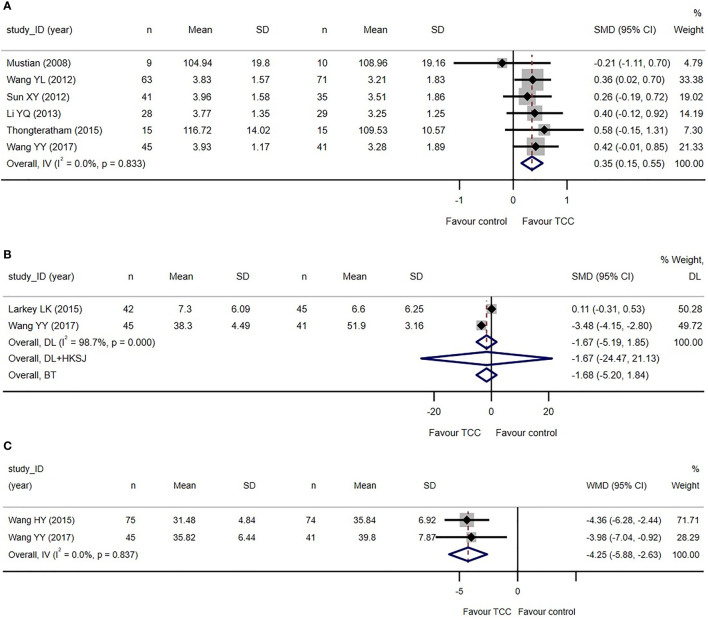
Primary outcomes of the systematic review of Tai Chi Chuan training on the QoL and psychological well-being in female patients with breast cancer. **(A)** TCC-based exercises are superior to the controls on quality of life. **(B)** No difference between TCC-based exercises and the controls on depression. **(C)** TCC-based exercises are superior to the controls on anxiety. The weights of trials were provided by random effect model applying DL *tau* ^2^ estimator. BT, Biggerstaff–Tweedie approximate Gamma model; DL, DerSimonian–Laird estimator of *tau* ^2^; DL+HKSJ, Hartung–Knapp-Sidik–Jonkman (HKSJ) variance correction to DerSimonian–Laird estimator of *tau* ^2^; IV, fixed effect inverse variance; SD, standard deviation; SMD, standardized mean difference; TCC, Tai Chi Chuan; IV, fixed effect inverse variance. The study ID consists of the first author’s surname, the capital initials of the first author’s first name, and the year the first report of the RCTs was published or submitted as dissertation.

Only two trials reported the total score of the QoL scales (67–69, 78), and another four trials (70, 71, 73, 83) applying WHOQOL-BREF reported the subjects’ assessment of their overall QoL (G1 question: How would you rate your quality of life)?. We used these data to perform a meta-analysis on the overall QoL and showed that TCC-based exercise was better than the control group (SMD=0.35, 95%CI: 0.15–0.55, *I* ^2 ^= 0, model: fixed, IV). See **Figure 3A**. We re-expressed the result as the units of FACT-B (version 4, 37 items), setting standard deviation (SD) as 20.19 points, which was the weighted average SD of baseline measures of two samples of a minimal important difference (MID) study of FACT-B (version 2, 35 items) (86). MID was set as 7–8 points increase (86, 87). The equivalent WMD was 7.07 points (> MID lower threshold).

Sensitivity analyses showed that the results of meta-analysis were stable. Subgroup analyses did not show that frequency and duration of TCC practice have moderating effect see **Supplementary Figure S1**.

There were two, two, and four trials, respectively, reporting the scores of each subscale of SF-36 (67–69, 76), FACT-B (72, 79), and WHOQOL-BREF (70, 71, 73, 83). In all WHOQOL-BREF subscales, meta-analyses showed that the two groups were different in health satisfaction (WMD=0.51, 95%CI: 0.21–0.80, *I* ^2 ^= 0, model: random, DL), physical health (WMD=2.19, 95%CI: 0.77–3.60, *I* ^2 ^= 54.9%, model: random, DL), psychological health (WMD=2.38, 95%CI: 1.36–3.39, *I* ^2 ^= 52.3%, model: random, DL), and social relationships (WMD=1.37, 95%CI: 0.38–2.35, *I* ^2 ^= 0, model: random, DL). In all SF-36 subscales, the meta-analysis showed that there are no differences between the two groups. In all FACT-B subscales, meta-analyses showed that the two groups were different in emotional well-being (WMD=2.80, 95%CI: 0.37–5.24, *I* ^2 ^= 89.3%, model: random, DL), functional well-being (WMD=3.18, 95%CI: 2.43–3.93, *I* ^2 ^= 0, model: random, DL) and breast cancer subscale (WMD=2.60, 95%CI: 1.77–3.43, *I* ^2 ^= 0, model: random, DL). All the differences between groups supported that TCC-based exercises are better than the controls. Another trial reported more comprehensive subcategory (physical health standardized and mental health standardized) scores in SF-36 and did not show differences between the two groups (75) see **Supplementary Figure S2**.

##### 3.4.1.2 Depression and anxiety

Two trials reported depression measured by Beck Depression Inventory (BDI) (74, 75) and Self-Rating Depression Scale (SDS) (74, 75, 83). The meta-analysis failed to detect the difference between the two groups (SMD=−1.67, 95%CI: −5.19–1.85, *I* ^2 ^= 98.7%, model: random, DL). Two trials reported anxiety measured by Self-Rating Anxiety Scale (SAS) (77, 83). The meta-analysis revealed that TCC-based exercises are better than the controls in improving anxiety (WMD=−4.25, 95%CI: −5.88 to −2.63, *I* ^2 ^= 0, model: fixed, IV). We did not set a MID for SAS because no studies have covered it yet see **Figures 3B, C**.

#### 3.4.2 Secondary outcomes

##### 3.4.2.1 Fatigue

**Figure 4:**
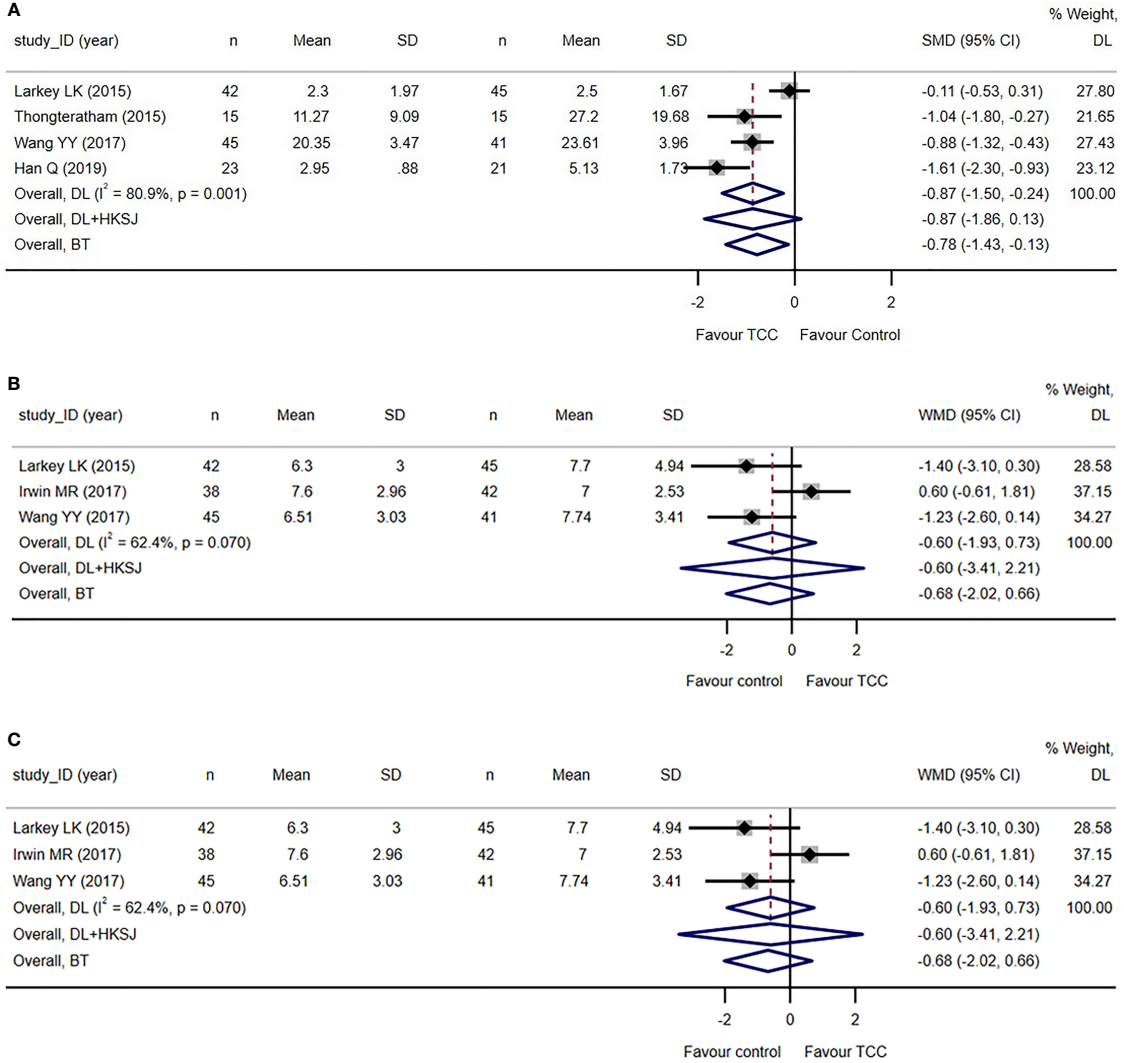
Secondary and *post-hoc* outcomes of the systematic review of Tai Chi Chuan training on the QoL and psychological well-being in female patients with breast cancer. **(A)** TCC-based exercises are superior to other interventions on fatigue when *tau* ^2^ was calculated by DL estimator in random effect model. Sensitivity analysis using DL + HKSJ as *tau* ^2^ estimator shows no difference between the two groups. **(B)** No difference between TCC-based interventions and other interventions on sleep quality. **(C)** TCC-based exercises are superior to the controls on shoulder function in breast cancer patients. The weights of trials were provided by random effect model applying DL *tau* ^2^ estimator. BT, Biggerstaff–Tweedie approximate Gamma model; DL, DerSimonian–Laird estimator of tau2; DL+HKSJ, Hartung–Knapp–Sidik–Jonkman (HKSJ) variance correction to DerSimonian–Laird estimator of *tau* ^2^; SD, standard deviation; SMD, standardized mean difference; TCC, Tai Chi Chuan. The study ID consists of the first author’s surname, the capital initials of the first author’s first name, and the year the first report of the RCTs was published or submitted as dissertation.

Five trials reported fatigue related to breast cancer (74, 75, 78, 83–85). Fatigue Symptom Inventory (FSI) (74, 75, 78) and Cancer Fatigue Scale (CFS) (83, 85) was used in two trials each, and the Revised Piper Fatigue Scale (PFS-R) were used in one trial (84). The meta-analysis of four trials (74, 75, 78, 83, 84) reporting total score of fatigue measuring scales showed that TCC-based exercises are superior to the controls (SMD=−0.87, 95%CI: −1.50 to −0.24, *I* ^2 ^= 80.9%, model: random, DL), which reached the standardized MID set for cancer-related fatigue of 0.70–0.89 points decrease (88) see **Figure 4A**.

However, the sensitive analysis using another *tau* ^2^ estimator in a random effect model showed that the result is unstable. Another sensitive analysis to detect the influence of each study by removing the study also shows that the result is unstable. Subgroup analysis shows that the frequency of TCC practice maybe a moderator in the effect size of fatigue measure and duration of TCC practice maybe not see **Supplementary Figure S3**.

Meta-analyses showed that TCC-based exercises are superior to the controls in physical fatigue (WMD=−2.76, 95%CI: −3.32 to −2.20, *I* ^2 ^= 0%, model: random, DL) and affective fatigue (WMD=−2.91, 95%CI: −5.04 to −0.79, *I* ^2 ^= 92.3%, model: random, DL) of CFS subscales see **Supplementary Figure S3**.

##### 3.4.2.2 Sleep quality

Three trials reported sleep quality measured by the Pittsburgh Sleep Quality Index (PSQI) (74, 75, 82, 83). The meta-analysis showed that there was no difference between TCC-based exercises and the controls (WMD=−0.60, 95%CI: −1.93–0.73, *I* ^2 ^= 62.4%, model: random, DL). See **Figure 4B**. The result is unstable by sensitive and subgroup analysis see **Supplementary Figure S4**.

##### 3.4.2.3 Cognitive function

Only one trial reported cognitive function measured by the Functional Assessment of Cancer therapy—Cognitive (FACT-COG, reported by participants) and the Wechsler Adult Intelligence Scale (WAIS-III, reported by investigators) (74, 75). The trial showed that the cognitive function measured by the two subscales of FACT-COG and the two subscales of WAIS-III had no statistical differences between the TCC-based exercise group and the control group.

##### 3.4.2.4 inflammatory cytokine

Only one trial reported inflammatory cytokines, showing that there were no differences between the two groups with regard to IL-2 (interleukin-2), IL-6 (interleukin-6), and IL-8 (interleukin-8) (67–69).

#### 3.4.3 Post-hoc analyses

##### 3.4.3.1 Shoulder function

Six trials reported shoulder function (70–72, 76, 79–81), of which four trials reported the total Constant–Murley score (70, 71, 76, 78, 81) and two trials reported the total Neer score (72, 79). The meta-analysis showed that TCC-based exercises outperformed the controls in improving overall shoulder function (SMD=1.12, 95%CI: 0.65–1.60, *I* ^2 ^= 85.0%, model: random, DL). See **Figure 4C**. We re-expressed the result as the units of Constant–Murley score, setting SD as 7.34 points, which was the weighted average SD of baseline measures of studies applying Constant–Murley score included in the meta-analysis. MID was set as 8.3 points increase, the median estimate with high credibility of Constant–Murley score (89). The equivalent WMD was 8.22 points (<MID).

Sensitive analysis shows that the result is unstable. Subgroup analyses did not suggest the presence of possible moderators. See **Supplementary Figure S5**.

Exploratory meta-analysis showed that TCC is better than the control group in all of the Constant–Murley subscales: pain (WMD=2.10, 95%CI: 0.10–4.09, *I* ^2 ^= 91.7%, model: random, DL), ADL (Activity of Daily Living) (WMD=3.11, 95%CI: 2.26–3.96, *I* ^2 ^= 29.4%, model: random, DL), ROM (Range of Motion) (WMD=2.93, 95%CI: 1.95–3.91, *I* ^2 ^= 14.9%, model: random, DL), and muscle strength (WMD=2.07, 95%CI: 1.11–3.04, *I* ^2 ^= 14.9%, model: random, DL) see **Supplementary Figure S6**.

##### 3.4.3.2 Time-effect analysis

Exploratory meta-regression analyses using a mixed effects model revealed that the cumulative weeks of practicing TCC had a significant effect on QoL, with longer cumulative practice leading to a greater SMD for QoL between the two groups (coefficient for cumulative practice weeks = 0.016/week; *p* = 0.006; 95%CI: 0.004–0.027). There was a similar time–response relationship for the SMD of shoulder function between the two groups (coefficient = 0.068/week; *p* < 0.001; 95%CI: 0.030–0.107).

### 3.5 Publication bias

We did not conduct Egger’s test, as there were no more than 10 trials included in any meta-analyses.

### 3.6 GRADE evidence of outcomes

The confidence to the results of the important outcomes was graded as very low certainty to moderate certainty. We did not downgrade the certainty of any outcomes in the domain of limitations and publication bias. We downgraded the certainty of all of the outcomes by one degree in the domain of indirectness because the meta analyses synthesized various comparisons. In the domain of Inconsistency and Imprecision, we downgraded the certainty by zero to two degrees. A summary of the finding table including all of these bodies of evidence is shown in **Table 1**.

**Table 1:** GRADE evidence profile of outcomes—summary of finding table of TCC-based exercise vs. non-TCC interventions for breast cancer.

Outcomes	Study design	Number of RCTs	Number of participants		Limitation	Inconsistency	Indirectness	Imprecision	Publication bias	Effect absolute (95%CI)	Certainty
			TCC	NT							
QOL	RCT	6	201	201	Not Serious	Not Serious	Serious^a^	Not Serious	Not Serious	SMD 0.35 higher (0.15 higher to 0.55 higher), Equivalent to a WMD of 7.07 points higher in the FACT-B	⨁⨁⨁◯ moderate
Depression	RCT	2	87	86	Not Serious	Very Serious^b^	Serious^a^	Very Serious^c^	Not Serious	SMD 1.67 lower (5.19 lower to 1.85 higher)	⨁◯◯◯ VERY LOW
Anxiety	RCT	2	120	115	Not Serious	Not Serious	Serious^a^	Not Serious	Not Serious	SMD 4.25 lower (5.88 lower to 2.63 lower)	⨁⨁⨁◯ moderate
Fatigue	RCT	4	125	122	Not Serious	Serious^d^	Serious^a^	Not Serious	Not Serious	SMD 0.87 lower (1.50 lower to 0.24 lower)	⨁⨁◯◯ LOW
Sleep quality	RCT	3	125	128	Not Serious	Not Serious	Serious^a^	Serious^e^	Not Serious	WMD 0.60 lower (1.93 lower to 0.73 higher)	⨁⨁◯◯ LOW
Shoulder function	RCT	6	278	272	Serious^f^	Serious^d^	Serious^a^	Not Serious	Not Serious	SMD 1.21 higher (0.65 higher to 1.60 higher), Equivalent to a WMD of 8.22 points higher in the Constant-Murley	⨁◯◯◯ VERY LOW

CI, confidence interval; NT, non-TCC interventions; QOL, quality of life; RCT, randomized controlled trial; SMD, standardized mean difference; TCC, Tai Chi Chuan; WMD, weighted mean difference.

a Multiple comparison types.

b Point estimates vary widely between studies, confidence intervals do not overlap, and heterogeneity I^2^ value is large.

c Meta-analysis sample size does not reach the optimal information size, 95%CI overlaps zero and both important benefit and harm included.

d Minimal overlap of 95%CI and heterogeneity I^2^ value is large.

e The 95%CI overlaps zero and both important benefit and harm included.

f High risk of bias with unstable effect estimates of sensitive analysis.

## 4 Discussion

In this study, we identified outcomes, including the most important QoL, typical symptoms of psychological distress (depression and anxiety), fatigue and sleep quality affecting physical and mental health, and cognitive levels affecting QoL in older patients, based on background study on the top concerns of breast cancer patients and the purpose of this study (15). Although shoulder function was not a concern for us at the beginning, we finally found that this outcome measure including shoulder pain is an important issue for breast cancer patients. Shoulder function (including localized pain) should be considered an outcome that is not related to overall self-perceived health (90), so we included shoulder function as the outcome of the *post-hoc* analysis. We did not analyze safety outcomes because such outcomes are rarely reported in TCC trials.

The results showed that TCC-based exercise was superior in improving QoL (statistically and clinically significant) and anxiety (statistically significant) in women with breast cancer compared to other currently available controls, with moderate-certainty evidence. TCC-based exercise was also shown to be superior to controls in improving fatigue (statistically and clinically significant), but the evidence was only low certainty. For other outcomes, meta-analyses failed to show a difference between TCC-based exercise and the control group, or the quality of the evidence was too low to make an informed judgment see **Table 1**.

Generally, this systematic review updates previous evidence on related topics to achieve the purpose of evaluating whether TCC-based exercise is beneficial for the QoL and psychosomatic symptoms in female patients with breast cancer. The included studies provided relatively broad answers to the review questions from different perspectives (e.g., different stages of breast cancer treatment, different types of controls, different cultural backgrounds) (67–85). We have some indicative evidence, but due to the scarcity of original TCC-related studies, we cannot yet give a certain answer on specific subgroups, control types, and specific outcome measures. Therefore, it is necessary to continue to update and refine the systematic review research on related topics.

In grading the outcome-centered evidence body, we adopted the GRADE system while consulting the embodied tools used by another team working on TCC to ensure transparency and reproducibility (91). One study showed good inter-rater reliability in the use of GRADE, and two individual raters can reliably assess the quality of evidence by GRADE (92). We believe that applying the same criteria for downgrading evidence can also be a good way to ensure reliability in the ratings of evidence for similar systematic reviews. In terms of methodological limitations (93), performance bias was not considered because personnel blinding in TCC studies were impractical. Careful and comprehensive criteria were used with respect to publication bias (94), imprecision (95), inconsistency (96), and indirectness (97) to ensure that certainty of evidence was not indiscriminately downgraded see **Supplementary Table 3**.

There may be some bias in the process of this systematic review. In the past decade, studies on TCC have come from all over the world, and many countries have a variety of native languages (50). Although we did not restrict the publication language of the original studies included, only reports published in Chinese or English were available in the databases that we searched, which may have biased the findings. Second, in this study, we did not contact the authors of the included studies for as much details as possible. This may allow us to underestimate the risk of bias of the original study and to overestimate the grade of evidence. Furthermore, the meta-analyses in this systematic review pooled several different comparison types. The underlying assumption was that practicing TCC had a clear advantage over other interventions that used as controls or that in combination with TCC in experimental group, so that the between-group differences were large enough to ignore statistical heterogeneity due to other factors. This assumption is based on the practice of previous systematic reviews of TCC in the treatment of breast cancer. However, it is clear that clinical heterogeneity remains, and the pooled results of this study are insufficient for making decision in the presence of previously uncompared interventions, such as yoga. That is why we downgraded the certainty of all of the outcomes by one degree in the domain of indirectness.

On the other hand, this study may also be biased by the trials included. First, the details of TCC practice are not reported in a standardized manner; this would be potential heterogeneity that could destabilize the synthetic results after the addition of new trials in the future. In addition, the methodological quality of the included trials was generally poor, placing a higher risk of bias in the synthesized results. The sample size of all included trials was small, which could lead to false positive results.

This study also has some methodological improvements compared to previous systematic reviews on the same topic. The first point is that this study places more emphasis on interpretation of the results. We reformulated SMDs to make it more intuitive and clarified the clinical value of the evidence by comparison with MID. We hope that the available indicative evidence will facilitate more primary research on TCC and interpretation that values clinical implications in primary and secondary research. The second point is that we adopted a more transparent and reproducible method of grading evidence, emphasizing the significance of the GRADE approach for grading and translation of evidence. Finally, This study was more cautious in including the original study and in performing the meta-analysis such as more sensitivity analysis.

In terms of results, our review included four more original study reports than the latest systematic review on the same topic published by Luo et al. The findings of our study on QoL, anxiety, fatigue, and shoulder function are similar to those of Luo et al. (58). Our study found no between-group differences in depression and sleep quality, which is similar to the findings of Liu et al. (57). However, our study was different from the review of Luo et al. in the grading of certainty of evidence in QoL, anxiety, and shoulder function. Because we were unable to know the thresholds or key factors at which it downgraded certainty of evidence in other reviews, the difference cannot be explained. The study by Luo et al. analyzed pain by synthesizing global pain from the QoL subscale with local pain from the shoulder function subscale. We suggest that the two types of pain are fundamentally different. The study of Luo et al. used TCC practice time as a factor for subgroup analysis, and we added practice frequency on this basis. We also attempted to find a linear corelation between the SMDs and cumulative TCC practice time by meta-regression as an exploration in *post-hoc* analysis (58).

Finally, from the perspective of promoting evidence dissemination and application, we suggest that for mind–body interventions such as TCC, understanding the factors and motivations that may affect the participation of breast cancer patients can be targeted to develop strategies to promote evidence translation (98, 99). It is very necessary for more female patients with breast cancer to improve their QoL through TCC and obtain social support (100).

## 5 Conclusion

Within the range of comparisons covered in this study, we believe that TCC-based exercise has potential advantage in improving QoL and psychological well-being of breast cancer patients. Practicing TCC can be time-accumulated beneficial for breast cancer patients from a short time after surgery to survival period. This conclusion should be used with caution given the risk of bias in the findings, possible adverse events, disputes over the interests and values of different patient groups, and other context-specific differences.

Future studies of TCC on breast cancer should pay more attention to outcomes that are important to patients and the reporting of intervention details. More alternative exercise interventions should be looked at and used as controls. Larger, well-designed and conducted randomized controlled trials with longer follow-up is warranted.

## Data availability statement

The original contributions presented in the study are included in the article/**Supplementary material**. Further inquiries can be directed to the corresponding author.

## Author contributions

JG put forward the idea of this systematic review and carried out the study design. FY played a coordinating and communicating role throughout the study. YS searched the clinical trial reports, and then, WL and JG conducted study selection, data extraction, and methodological quality assessment independently. QW and JW analyzed the data. Finally, WL assessed the certainty of the evidence and interpreted the data before writing this systematic review. The level of evidence and interpretations were agreed with by all authors. The corresponding author JG can be contacted for any process information request. The authors declare that they have no competing interests.
