# Supplementary material for: Effects of Tai Chi Chuan training on the QoL and psychological well-being in female patients with breast cancer: a systematic review of randomized controlled trials
Source: Front Oncol. 2023 May 1;13:1143674. doi: 10.3389/fonc.2023.1143674 (PMC10183581; doi:10.3389/fonc.2023.1143674)
Supplement: Supplementary file 1 [file DataSheet_1.docx]

Supplementary Material

Effects of Tai Chi Chuan training on the QoL and psychological well-beings in women patients with Breast Cancer: A Systematic Review of Randomized Controlled Trials

Wenyuan Li^1,2,3^, Fengming You^1,3^, Qiaoling Wang^1,3^, Yifeng Shen^1^, Jundong Wang^1^, and Jing Guo^1,*^

1 Hospital of Chengdu University of Traditional Chinese Medicine, Chengdu, Sichuan Province, P. R. China.

2 Evidence Based Traditional Chinese Medicine Center of Sichuan Province, Chengdu, Sichuan Province, P. R. China.

3 TCM Regulating Metabolic Diseases Key Laboratory of Sichuan Province, Chengdu, Sichuan Province, P. R. China.

*** Correspondence:**Jing Guo
guojing19910307@sina.com

## **Supplementary Figures**


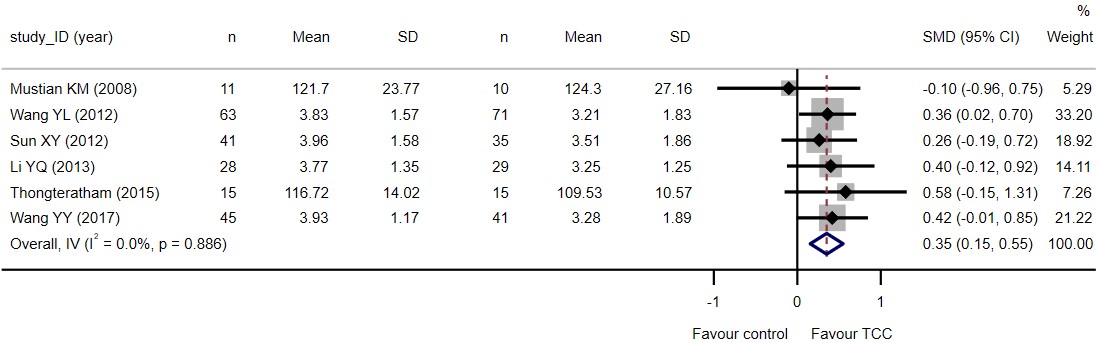


(A)


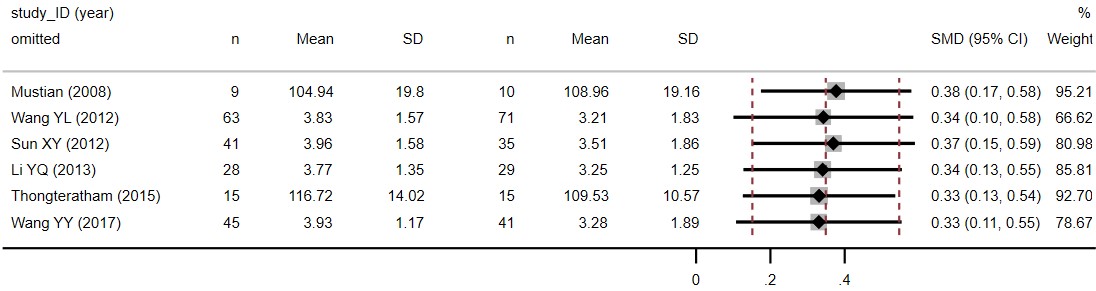


(B)


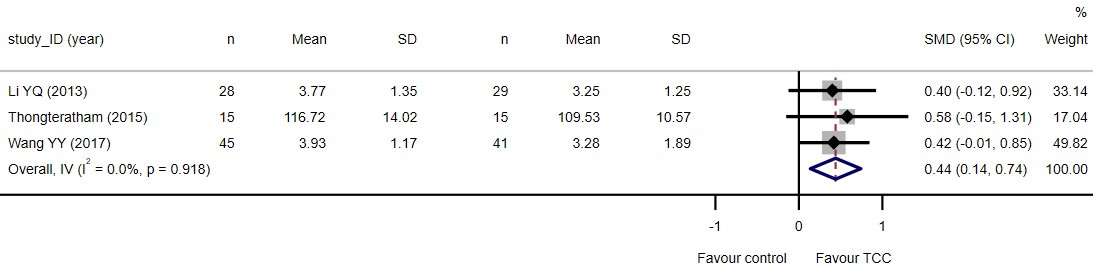


(C)


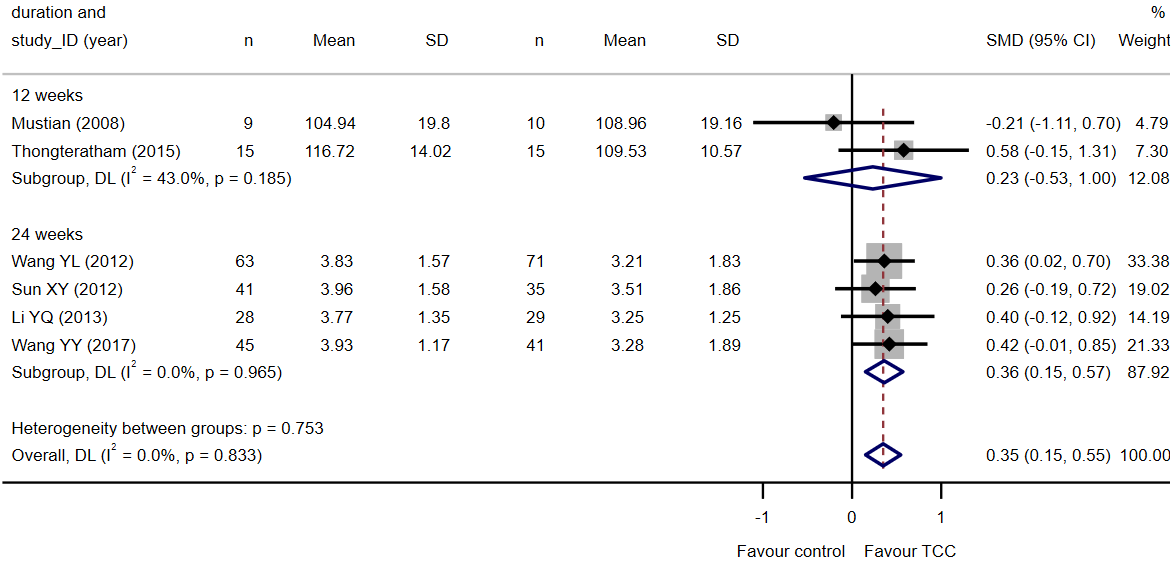


(D)


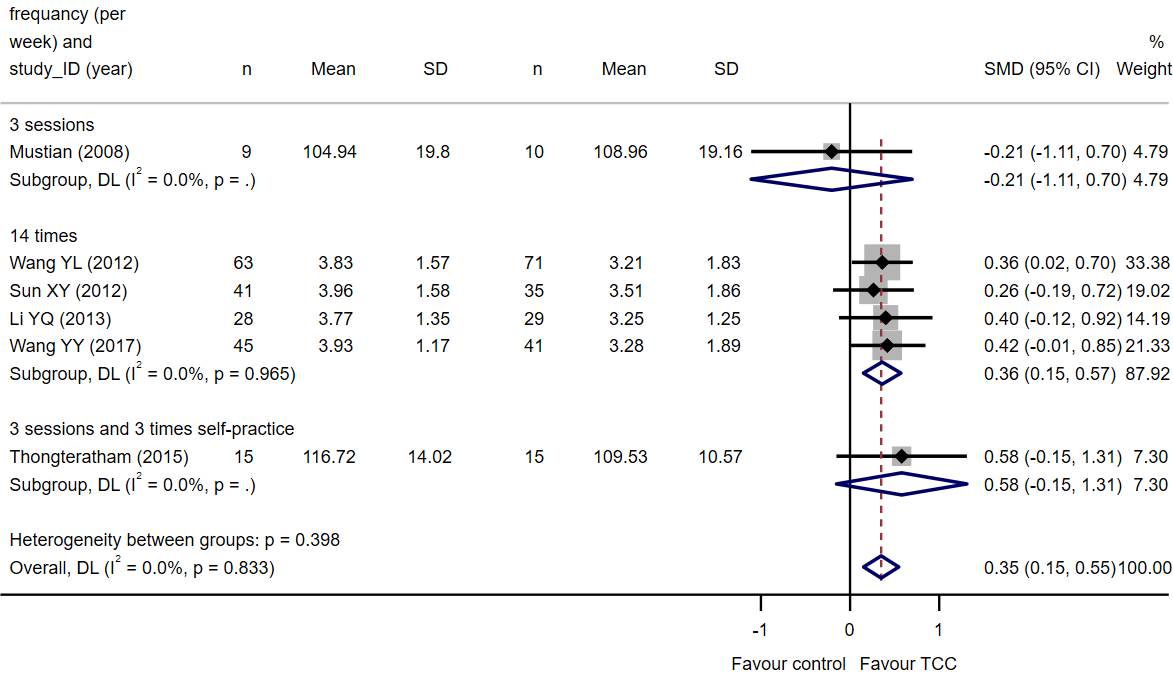


(E)

Figure 1: (A) Sensitive analysis replacing the measure FACIT-F with SF-36 for QoL in Mustian KM (2008) shows that the result is stable. (B) Sensitive analysis to detect the influence of each study by removing the study shows that the result is stable. (C) Sensitive analysis by removing studies with low methodological quality shows that the result is stable. (D) subgroup analysis by duration of TCC practice shows no difference between the two groups. (E) subgroup analysis by frequency of TCC practice shows no difference between the two groups. The weights of trials of meta-analyses employing random effect models were provided by DL tau^2^ estimator. DL: DerSimonian-Laird estimator of tau^2^; IV: Fixed effect inverse-variance; SD: Standard Deviation; SMD: Standardized mean difference; TCC: Tai Chi Chuan.


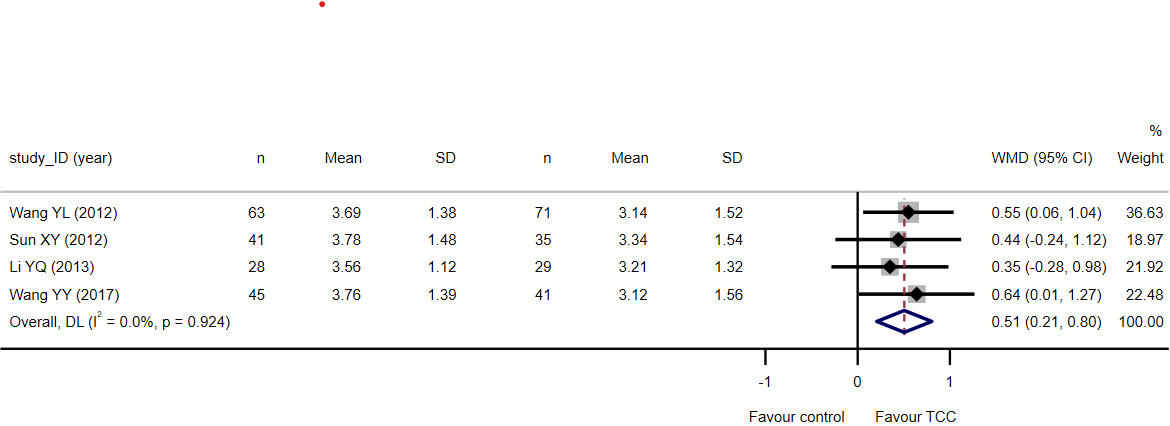


(A) WHOQOL-BREF: Health satisfaction


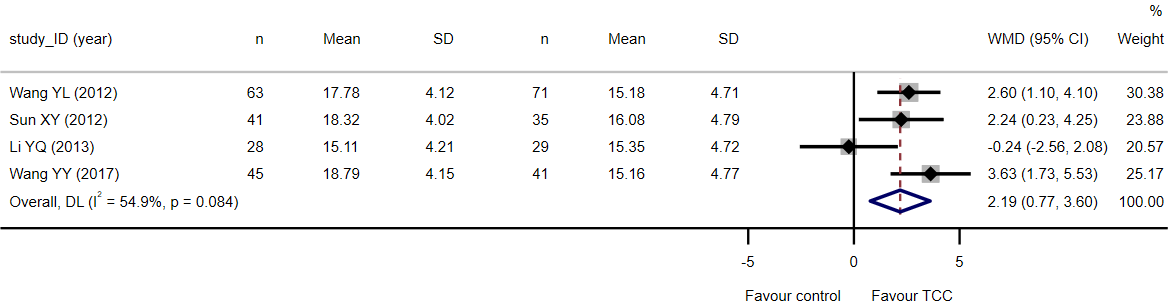


(B) WHOQOL-BREF: Physical health


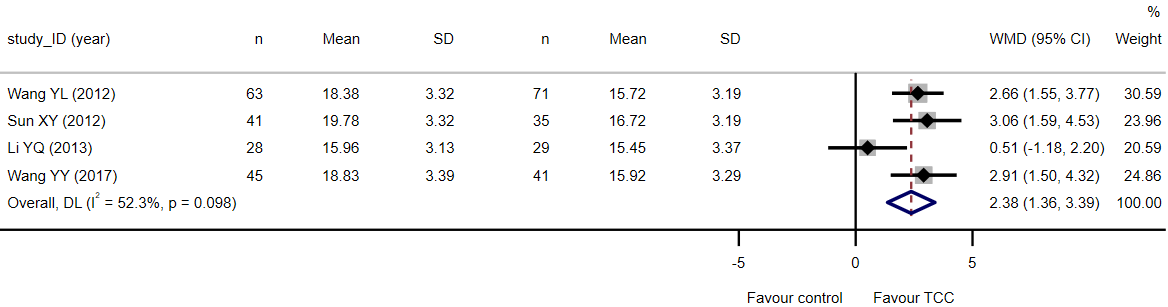


(C) WHOQOL-BREF: Psychological


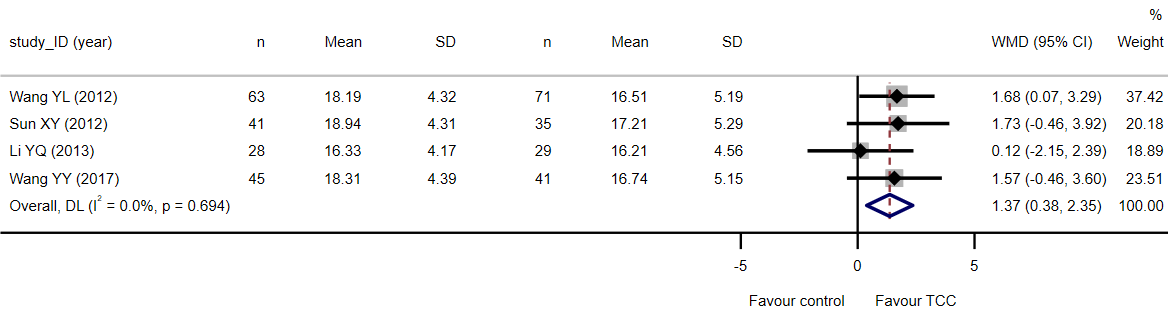


(D) WHOQOL-BREF: Social relationships


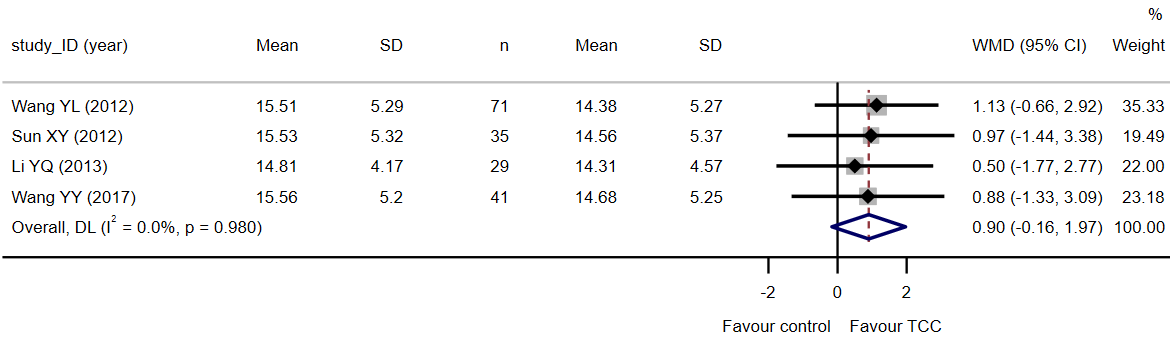


(E) WHOQOL-BREF: Environment


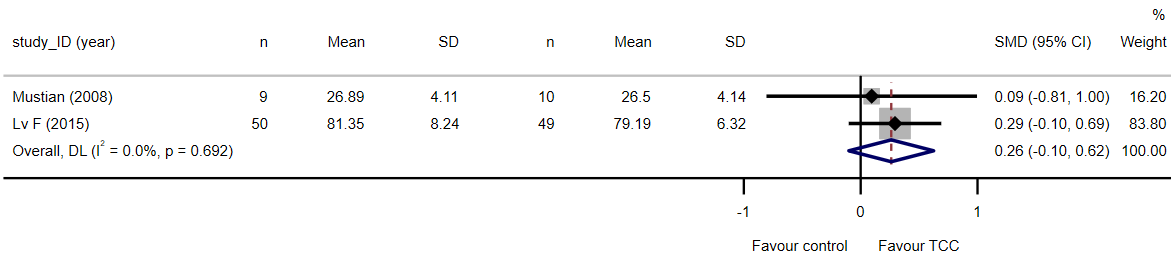


(F) SF-36: Physical functioning


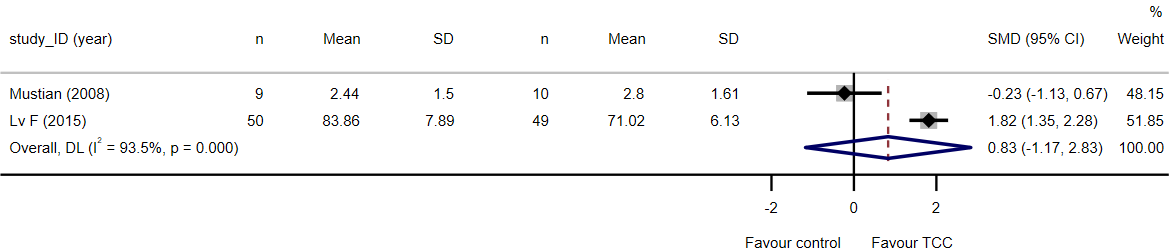


(G) SF-36: Role limitations due to physical health


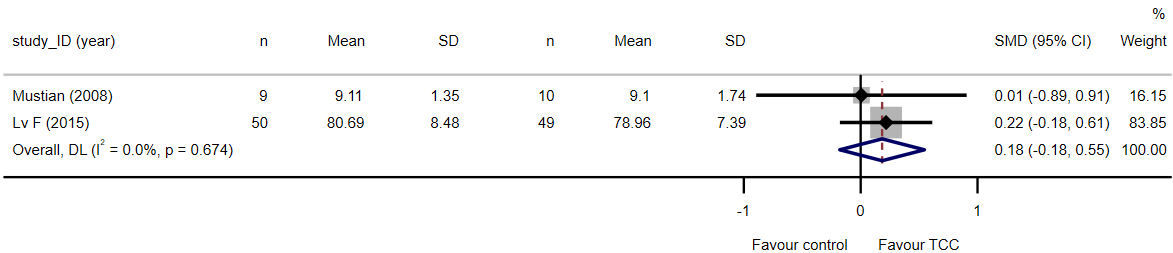


(H) SF-36: Pain


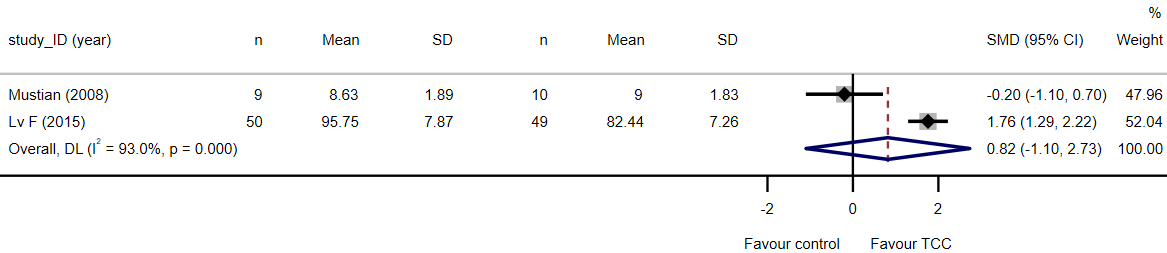


(I) SF-36: Social functioning


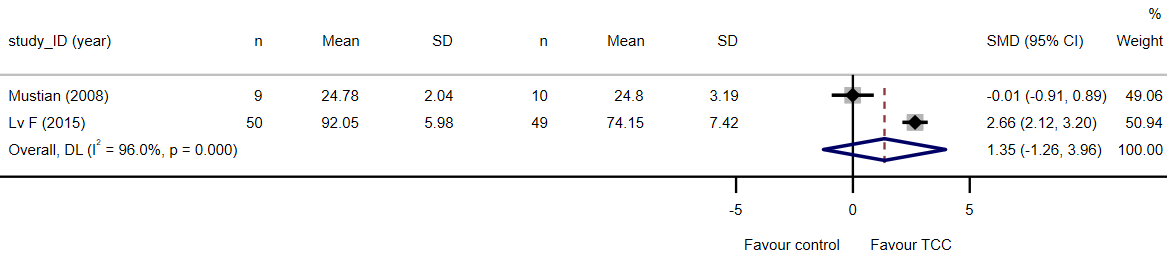


(J) SF-36: Emotional well-being


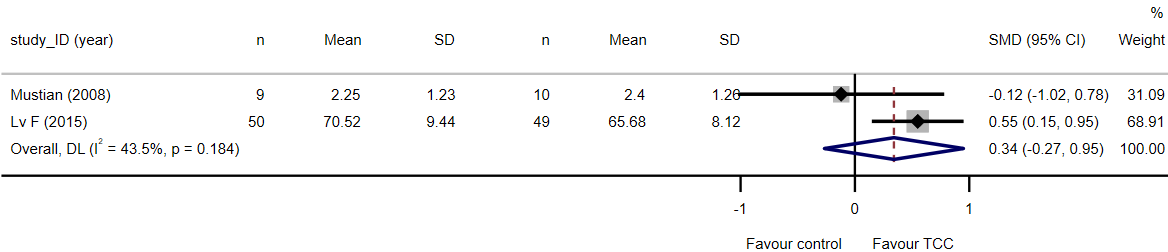


(K) SF-36: Role limitations due to emotional problems


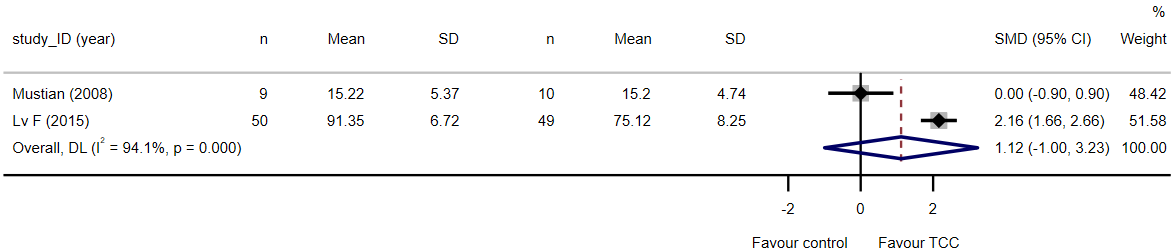


(L) SF-36: Energy/fatigue


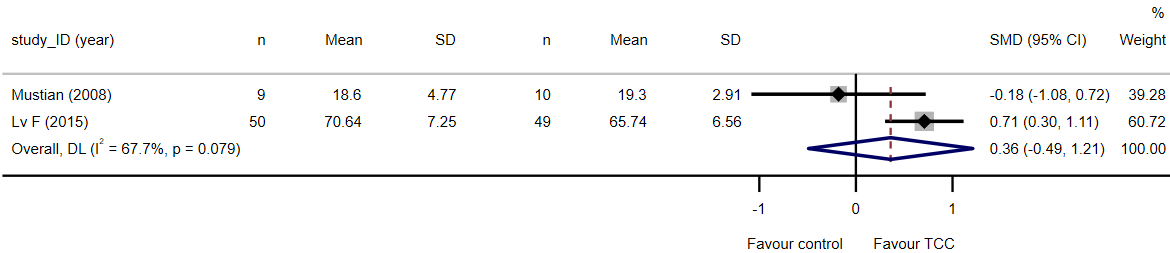


(M) SF-36: General health


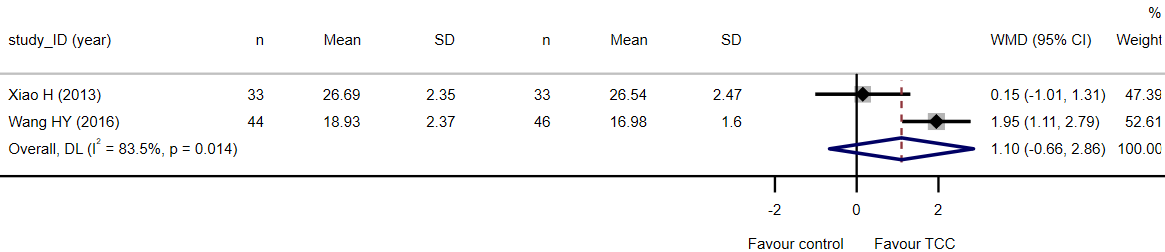


(N)FACT-B: Physical well-being


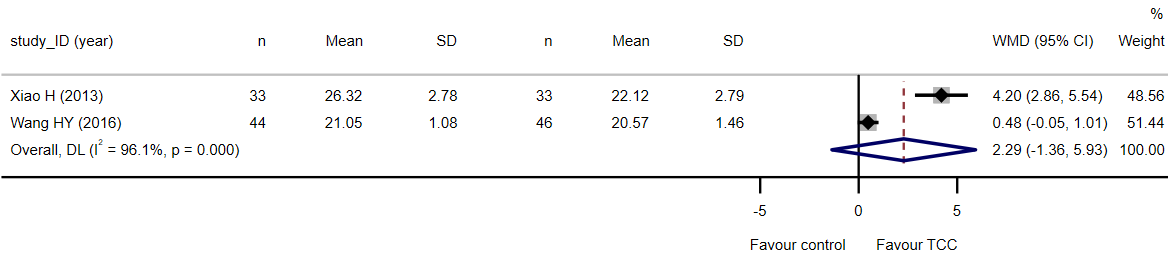


## (O) FACT-B: Social/Family well-Being


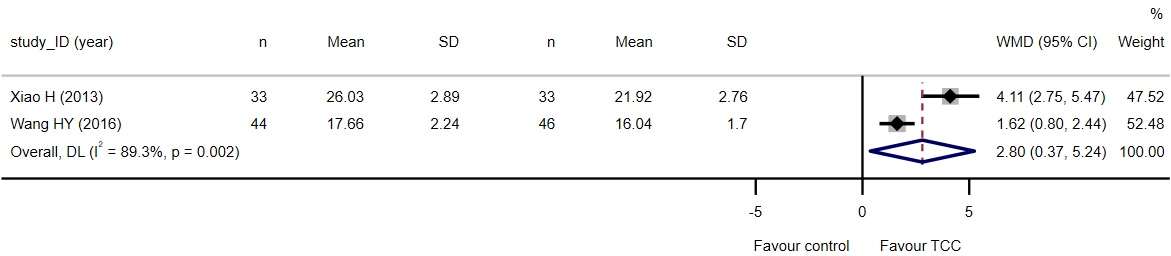


(P) FACT-B: Emotional well-Being


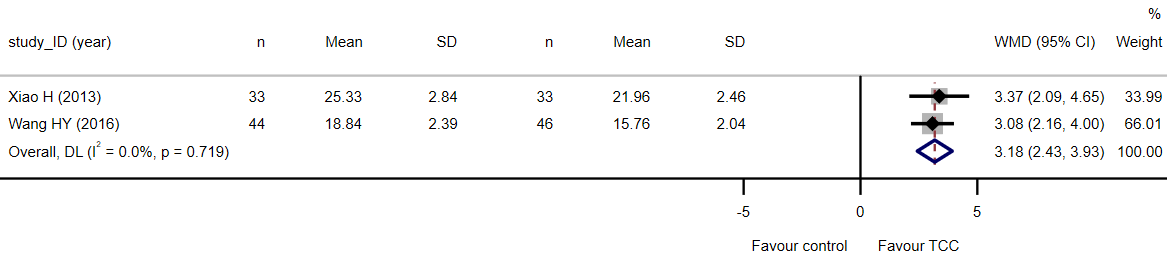


(Q) FACT-B: Functional well-Being


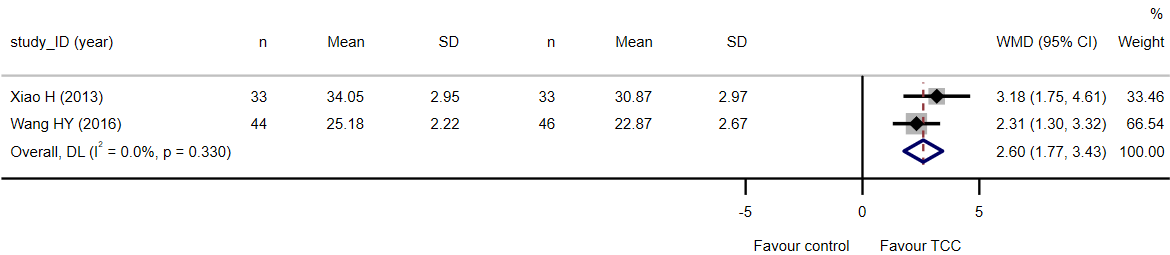


(R) FACT-B: Breast cancer subscale

Figure 2: Meta-analysis of subscales shows that TCC-based exercises are superior to the controls in (A) health satisfaction, (B) physical health, (C) psychological health and (D) social relationships of WHOQOL-BREF; (P) emotional well-being, (Q) functional well-being and (R) breast cancer subscale of FACT-B. The weights of trials of meta-analyses employing random effect models were provided by DL tau^2^ estimator. DL: DerSimonian-Laird estimator of tau2; SD: Standard Deviation; SMD: Standardized mean difference; TCC: Tai Chi Chuan; WMD: weighted mean difference.


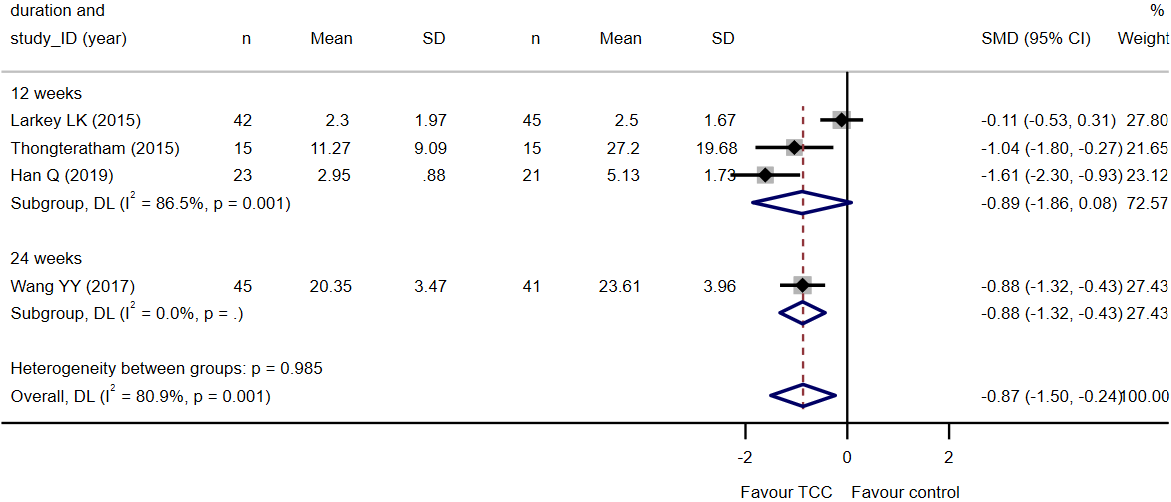


(A)


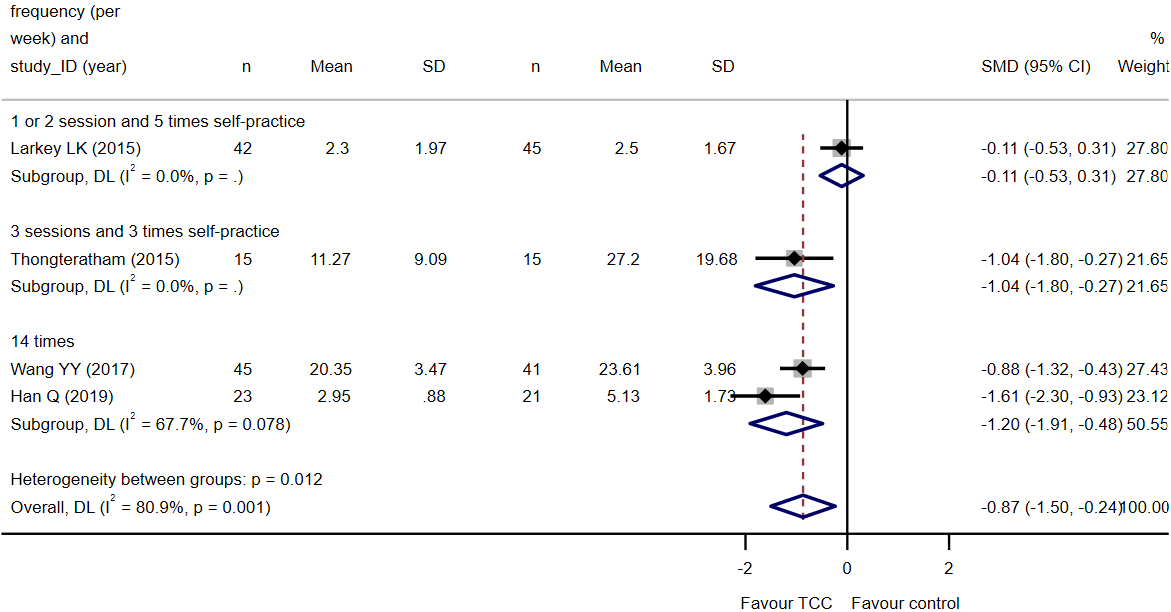


(B)


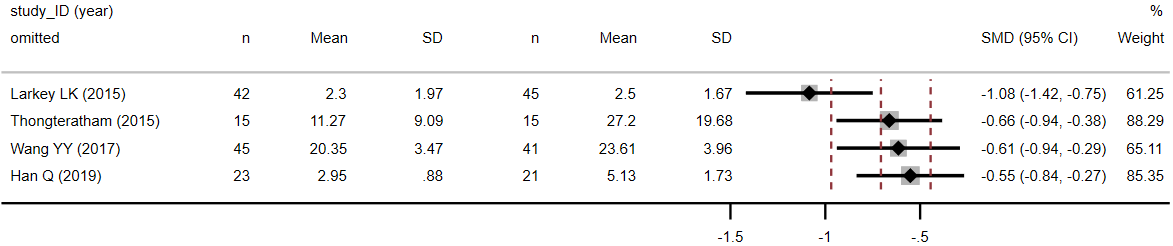


(C)


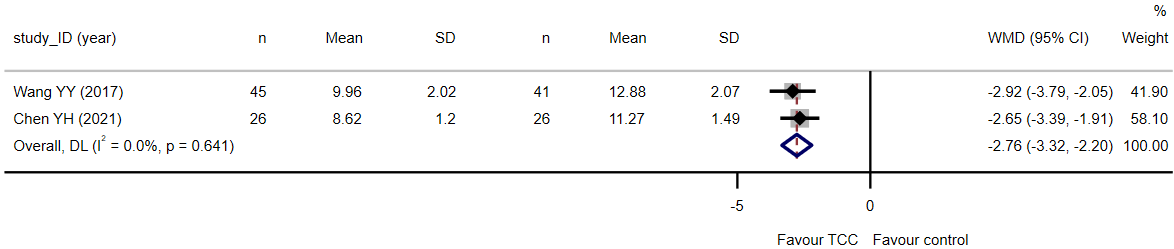


(D) Physical fatigue


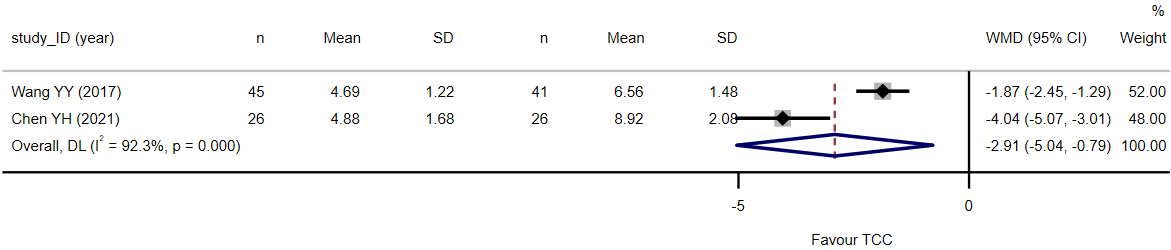


(E) Affective fatigue


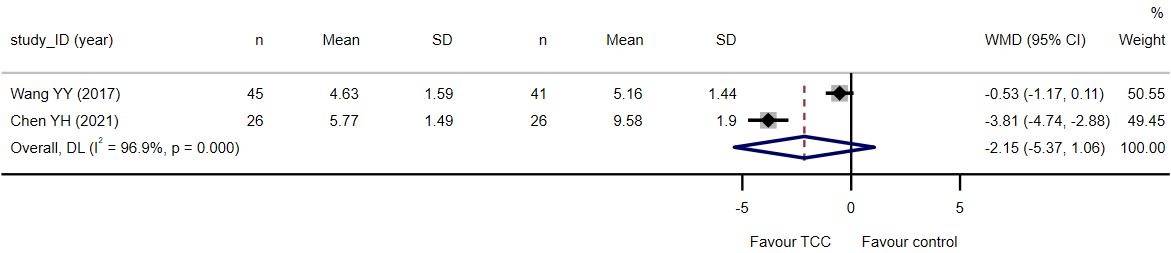


(F) Cognitive fatigue

Figure 3: (A) Subgroup analysis by duration of TCC practice shows no difference on fatigue between the two groups. (B) Subgroup analysis by frequency of TCC practice and (C) sensitive analysis to detect the influence of each study by removing the study shows that the result is unstable. Meta-analysis of subscales of CFS shows that TCC-based exercises are superior to the controls in (D) physical fatigue and (E) affective fatigue of cancer fatigue scale. The weights of trials of meta-analyses employing random effect models were provided by DL tau^2^ estimator. DL: DerSimonian-Laird estimator of tau^2^; SD: Standard Deviation; SMD: Standardized mean difference; TCC: Tai Chi Chuan; WMD: weighted mean difference.


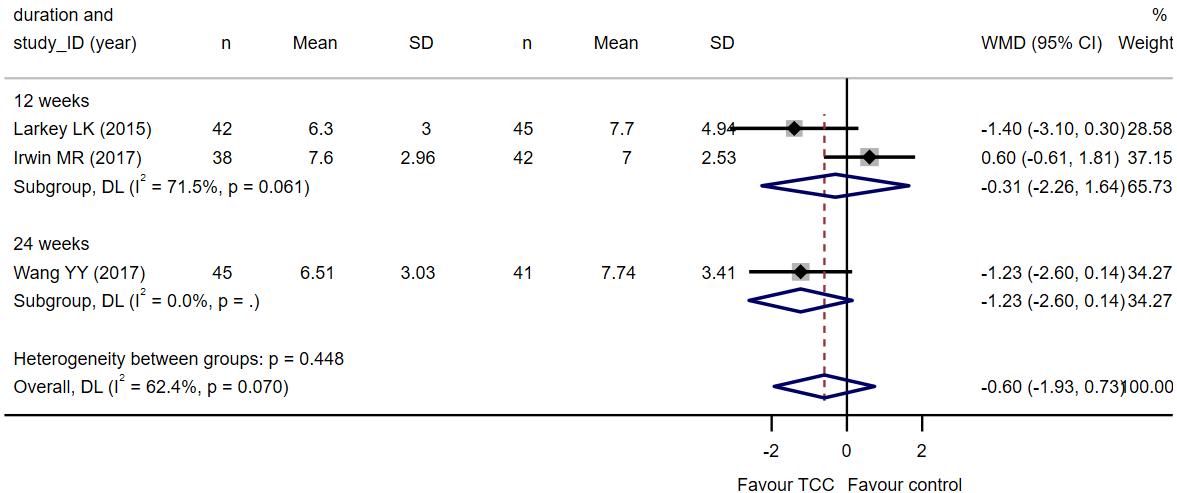


(A)


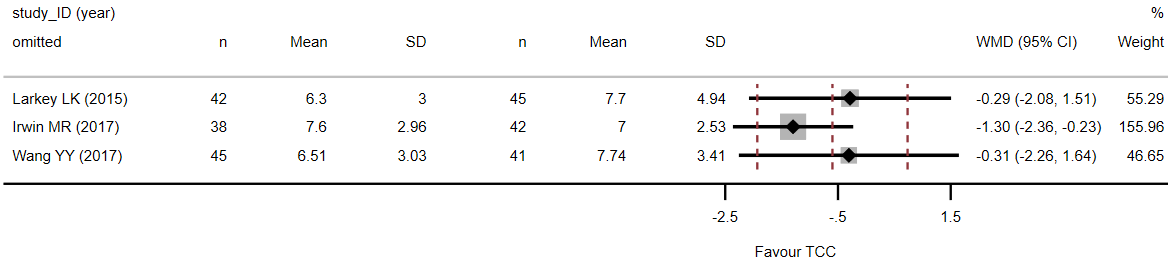


(B)

Figure 4: (A) Subgroup analyses by duration of TCC practice shows no difference on sleep quality between the two groups. (B) Sensitive analysis to detect the influence of each study by removing the study shows that the result is unstable. The weights of trials of meta-analyses employing random effect models were provided by DL tau^2^ estimator. DL: DerSimonian-Laird estimator of tau^2^; SD: Standard Deviation; TCC: Tai Chi Chuan; WMD: weighted mean difference.


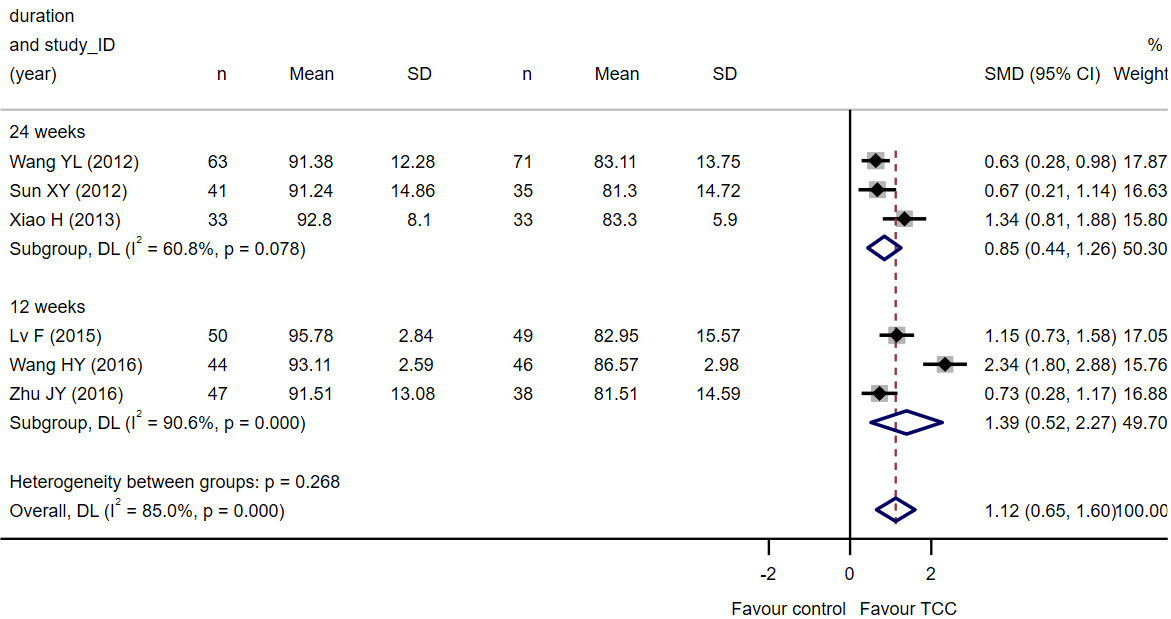


(A)


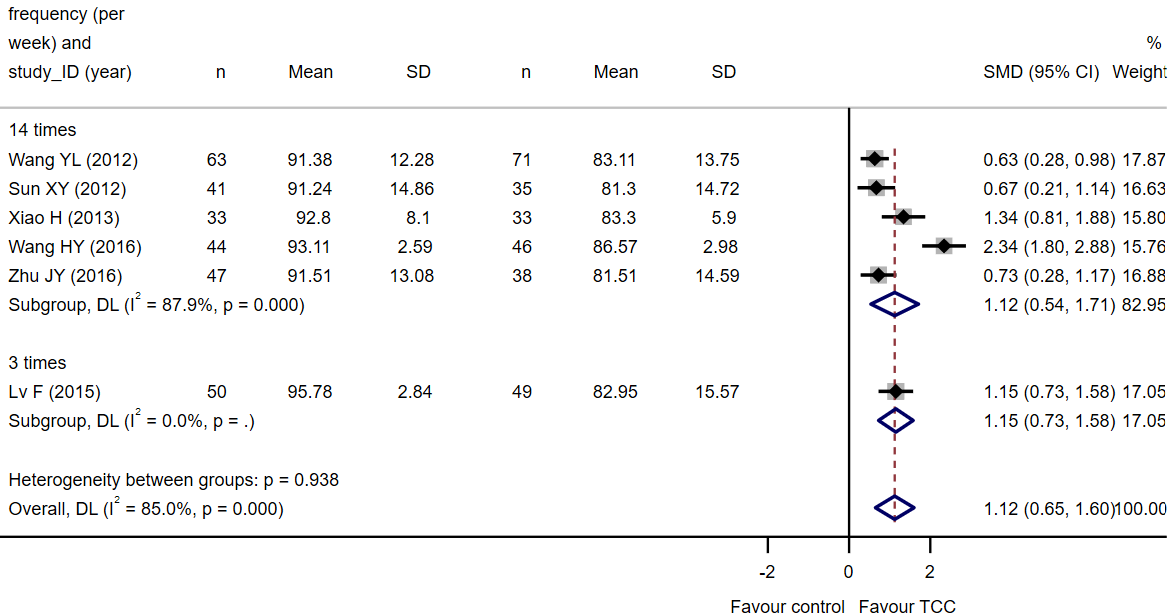


(B)


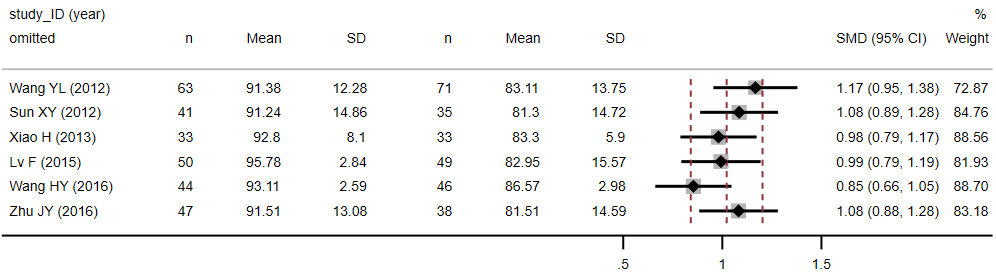


(C)

Figure 5: (A) Subgroup analyses by duration of TCC practice shows no difference on shoulder function between the two groups. (B) Subgroup analyses by frequency of TCC practice also shows no difference on shoulder function between the two groups. (C) Sensitive analysis to detect the influence of each study by removing the study shows that the result is unstable. The weights of trials of meta-analyses employing random effect models were provided by DL tau^2^ estimator. DL: DerSimonian-Laird estimator of tau^2^; SD: Standard Deviation; SMD: Standardized mean difference; TCC: Tai Chi Chuan.


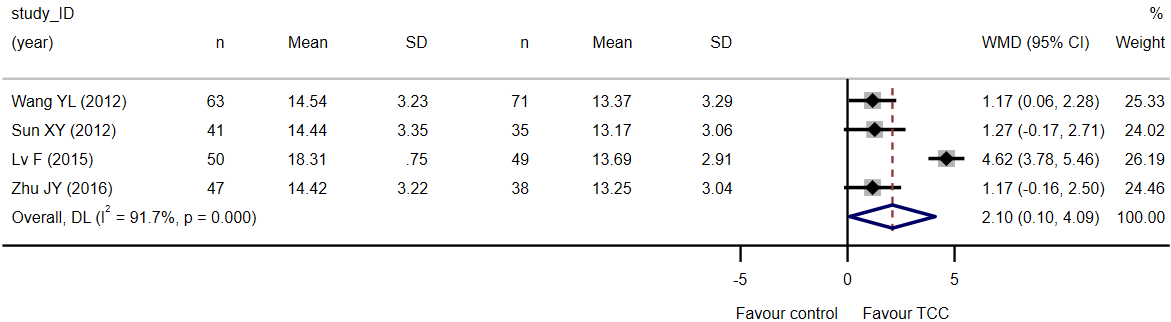


(A) Constant-Murley: pain


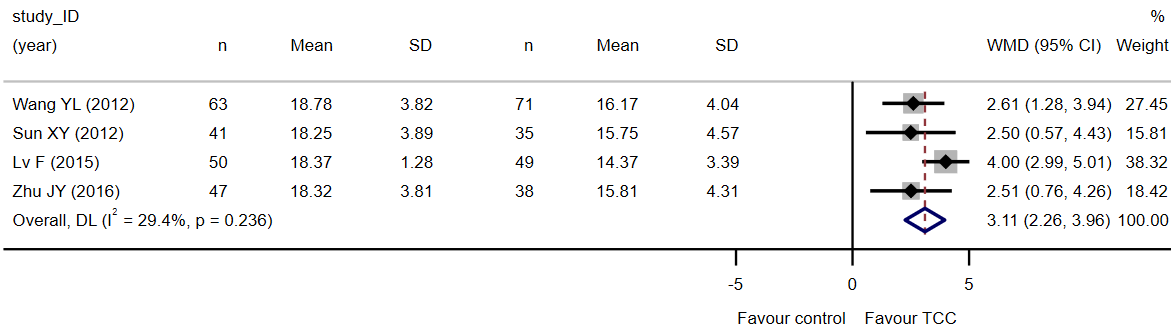


(B) Constant-Murley: ADL


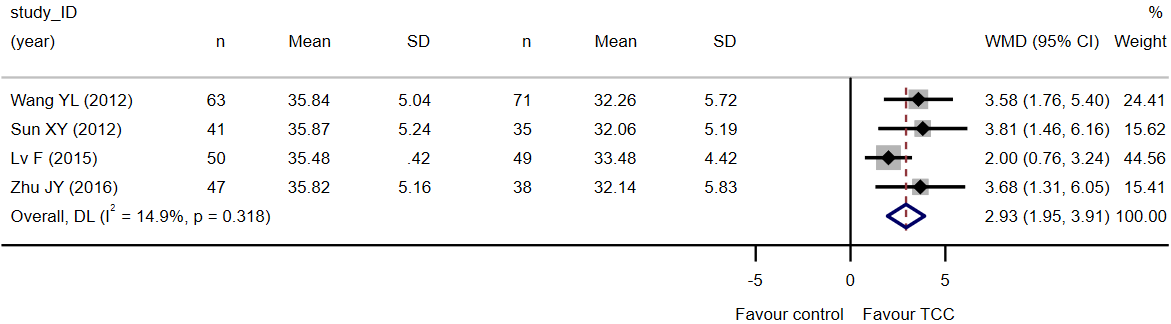


(C) Constant-Murley: ROM


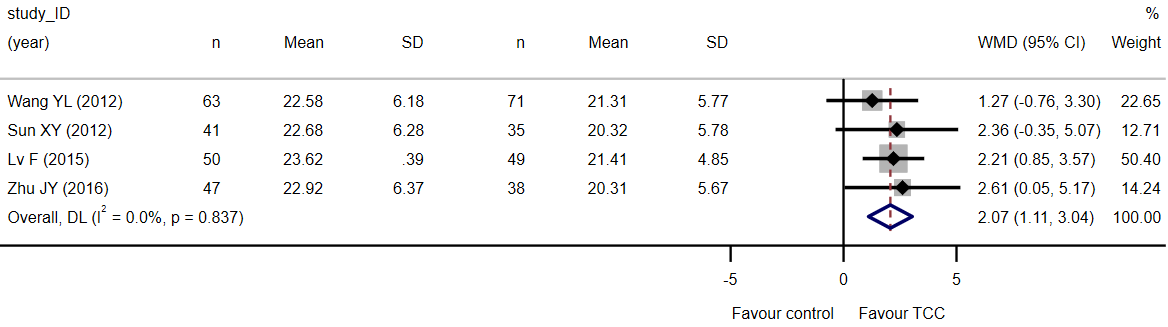


(D) Constant-Murley: muscle strength

Figures 6: Meta-analysis of subscales shows that TCC-based interventions are superior to other interventions in (A) pain, (B) ADL, (C) ROM and muscle (D) strength of Constant-Murley. The weights of trials of meta-analyses employing random effect models were provided by DL tau2 estimator. ADL: activities of daily living; DL: DerSimonian-Laird estimator of tau^2^; ROM: Range of motion; SD: Standard Deviation; TCC: Tai Chi Chuan; WMD: weighted mean difference.

## **Supplementary Tables**

Table 1. PRISMA checklist 2020

| Section and Topic | Item # | Checklist item | Location where item is reported |
| --- | --- | --- | --- |
| TITLE | | |  |
| Title | 1 | Identify the report as a systematic review. | Page 1. |
| ABSTRACT | | |  |
| Abstract | 2 | See the PRISMA 2020 for Abstracts checklist. | Line 17 to 41. |
| INTRODUCTION | | |  |
| Rationale | 3 | Describe the rationale for the review in the context of existing knowledge. | Line 43 to 98. |
| Objectives | 4 | Provide an explicit statement of the objective(s) or question(s) the review addresses. | Line 99 to 108. |
| METHODS | | |  |
| Eligibility criteria | 5 | Specify the inclusion and exclusion criteria for the review and how studies were grouped for the syntheses. | Line 129 to 152. |
| Information sources | 6 | Specify all databases, registers, websites, organisations, reference lists and other sources searched or consulted to identify studies. Specify the date when each source was last searched or consulted. | Line 116 to 122. |
| Search strategy | 7 | Present the full search strategies for all databases, registers and websites, including any filters and limits used. | Line 123 to 128; Supplementary material table 2. |
| Selection process | 8 | Specify the methods used to decide whether a study met the inclusion criteria of the review, including how many reviewers screened each record and each report retrieved, whether they worked independently, and if applicable, details of automation tools used in the process. | Line 154 to 155; Line 172 to 175. |
| Data collection process | 9 | Specify the methods used to collect data from reports, including how many reviewers collected data from each report, whether they worked independently, any processes for obtaining or confirming data from study investigators, and if applicable, details of automation tools used in the process. | Line 156 to 163; Line 172 to 175. |
| Data items | 10a | List and define all outcomes for which data were sought. Specify whether all results that were compatible with each outcome domain in each study were sought (e.g. for all measures, time points, analyses), and if not, the methods used to decide which results to collect. | Line 144 to 148; Line 181 to 182; Line 186 to 187. |
|  | 10b | List and define all other variables for which data were sought (e.g. participant and intervention characteristics, funding sources). Describe any assumptions made about any missing or unclear information. | Line 156 to 163. |
| Study risk of bias assessment | 11 | Specify the methods used to assess risk of bias in the included studies, including details of the tool(s) used, how many reviewers assessed each study and whether they worked independently, and if applicable, details of automation tools used in the process. | Line 166 to 171 |
| Effect measures | 12 | Specify for each outcome the effect measure(s) (e.g. risk ratio, mean difference) used in the synthesis or presentation of results. | Line 181 to 186. |
| Synthesis methods | 13a | Describe the processes used to decide which studies were eligible for each synthesis (e.g. tabulating the study intervention characteristics and comparing against the planned groups for each synthesis (item #5)). | Line 177 to 181 |
|  | 13b | Describe any methods required to prepare the data for presentation or synthesis, such as handling of missing summary statistics, or data conversions. | Line 164; Line 181. |
|  | 13c | Describe any methods used to tabulate or visually display results of individual studies and syntheses. | Line 194. |
|  | 13d | Describe any methods used to synthesize results and provide a rationale for the choice(s). If meta-analysis was performed, describe the model(s), method(s) to identify the presence and extent of statistical heterogeneity, and software package(s) used. | Line 181; Line 188 to 194. |
|  | 13e | Describe any methods used to explore possible causes of heterogeneity among study results (e.g. subgroup analysis, meta-regression). | Line 196 to 197. |
|  | 13f | Describe any sensitivity analyses conducted to assess robustness of the synthesized results. | Line 197 to 201. |
| Reporting bias assessment | 14 | Describe any methods used to assess risk of bias due to missing results in a synthesis (arising from reporting biases). | Line 208 to 209. |
| Certainty assessment | 15 | Describe any methods used to assess certainty (or confidence) in the body of evidence for an outcome. | Line 211 to 216; Supplementary material table 3. |
| RESULTS | | |  |
| Study selection | 16a | Describe the results of the search and selection process, from the number of records identified in the search to the number of studies included in the review, ideally using a flow diagram. | Line 219 to 229; Figure 1. |
|  | 16b | Cite studies that might appear to meet the inclusion criteria, but which were excluded, and explain why they were excluded. | Line 230 to 232; Supplementary material table 4. |
| Study characteristics | 17 | Cite each included study and present its characteristics. | Line 234 to 258; Supplementary material table 4. |
| Risk of bias in studies | 18 | Present assessments of risk of bias for each included study. | Line 260 to 280; Figure 2. |
| Results of individual studies | 19 | For all outcomes, present, for each study: (a) summary statistics for each group (where appropriate) and (b) an effect estimate and its precision (e.g. confidence/credible interval), ideally using structured tables or plots. | Figure 3 to 4; Supplementary material figures. |
| Results of syntheses | 20a | For each synthesis, briefly summarise the characteristics and risk of bias among contributing studies. | Figure 3 to 4. |
|  | 20b | Present results of all statistical syntheses conducted. If meta-analysis was done, present for each the summary estimate and its precision (e.g. confidence/credible interval) and measures of statistical heterogeneity. If comparing groups, describe the direction of the effect. | Line 283 to 379. |
|  | 20c | Present results of all investigations of possible causes of heterogeneity among study results. | Line 283 to 379. |
|  | 20d | Present results of all sensitivity analyses conducted to assess the robustness of the synthesized results. | Line 283 to 379. |
| Reporting biases | 21 | Present assessments of risk of bias due to missing results (arising from reporting biases) for each synthesis assessed. | Line 380 to 381 |
| Certainty of evidence | 22 | Present assessments of certainty (or confidence) in the body of evidence for each outcome assessed. | Line 382 to 388; Table 1. |
| DISCUSSION | | |  |
| Discussion | 23a | Provide a general interpretation of the results in the context of other evidence. | Line 390 to 405. |
|  | 23b | Discuss any limitations of the evidence included in the review. | Line 439 to 444. |
|  | 23c | Discuss any limitations of the review processes used. | Line 424 to 438. |
|  | 23d | Discuss implications of the results for practice, policy, and future research. | Line 406 to 423; Line 454 to 481. |
| OTHER INFORMATION | | |  |
| Registration and protocol | 24a | Provide registration information for the review, including register name and registration number, or state that the review was not registered. | Line 22; Line 111. |
|  | 24b | Indicate where the review protocol can be accessed, or state that a protocol was not prepared. | Line 111 to 112. |
|  | 24c | Describe and explain any amendments to information provided at registration or in the protocol. | Line 146 to 148. |
| Support | 25 | Describe sources of financial or non-financial support for the review, and the role of the funders or sponsors in the review. | Line 491 to 493. |
| Competing interests | 26 | Declare any competing interests of review authors. | Line 489. |
| Availability of data, code and other materials | 27 | Report which of the following are publicly available and where they can be found: template data collection forms; data extracted from included studies; data used for all analyses; analytic code; any other materials used in the review. | Line 488 to 489. |

Table 2. Details of search strategy

| Four English medical databases: Cochrane Library, PubMed, EMBASE, Web of science. |
| --- |
| Four Chinese medical databases: China National Knowledge Infrastructure Database (CNKI), Wan Fang Database, Sinomed, VIP Chinese Science and Technology Periodical Database (VIP). |
| Overall strategy:  (“Tai Chi Chuan” OR “Tai Chi” OR “Tai Ji” OR “Tai Ji Quan” OR “Taijiquan” OR “Taiji” OR “Tai-ji”) AND (“breast cancer” OR “breast carcinoma” OR “breast neoplasm” OR “breast tumor”) AND “random∗” |
| Strategy for Cochrane Library:  #1 MeSH descriptor: [Breast Neoplasms] explode all trees  #2 ('breast cancer'):ti,ab,kw OR ('breast carcinoma'):ti,ab,kw OR ('breast neoplasm'):ti,ab,kw OR ('breast tumor'):ti,ab,kw in Trials  #3 #1 OR #2  #4 MeSH descriptor: [Tai Ji] explode all trees  #5 ('Tai Chi Chuan'):ti,ab,kw OR ('Tai Chi'):ti,ab,kw OR ('Tai Ji'):ti,ab,kw OR ('Tai Ji Quan'):ti,ab,kw OR ('Taijiquan'):ti,ab,kw OR ('Taiji'):ti,ab,kw OR ('Tai-ji'):ti,ab,kw  #6 #4 OR #5  #7 random*  #8 #3 AND #6 AND #7 |
| Strategy for Pubmed:  ("breast neoplasms"[MeSH Terms] OR ("breast cancer"[Title/Abstract] OR "breast carcinoma"[Title/Abstract] OR "breast neoplasm"[Title/Abstract] OR "breast tumor"[Title/Abstract])) AND ("tai ji"[MeSH Terms] OR ("tai chi chuan"[Title/Abstract] OR "tai chi"[Title/Abstract] OR "tai ji"[Title/Abstract] OR "tai ji quan"[Title/Abstract] OR "taijiquan"[Title/Abstract] OR "taiji"[Title/Abstract] OR "tai ji"[Title/Abstract])) AND "random*"[All Fields] |
| Strategy for Embase:  ('breast neoplasms'/exp OR 'breast neoplasms' OR 'breast cancer':ti,ab,kw OR 'breast carcinoma':ti,ab,kw OR 'breast neoplasm':ti,ab,kw OR 'breast tumor':ti,ab,kw) AND ('tai ji'/exp OR 'tai ji' OR 'tai chi chuan':ti,ab,kw OR 'tai chi':ti,ab,kw OR 'tai ji':ti,ab,kw OR 'tai ji quan':ti,ab,kw OR 'taijiquan':ti,ab,kw OR 'taiji':ti,ab,kw OR 'tai-ji':ti,ab,kw) AND random* |
| Strategy for Web of science:  ((TS=('breast neoplasms' OR 'breast neoplasms' OR 'breast cancer' OR 'breast carcinoma' OR 'breast neoplasm' OR 'breast tumor')) AND TS=('tai ji' OR 'tai ji' OR 'tai chi chuan' OR 'tai chi' OR 'tai ji' OR 'tai ji quan' OR 'taijiquan' OR 'taiji' OR 'tai-ji')) AND TS=(random*) |
| Strategy for APA PsycInfo and Psychology and Behavioral Sciences Colletion:  AB ( AB “Tai Chi Chuan” OR “Tai Chi” OR “Tai Ji” OR “Tai Ji Quan” OR “Taijiquan” OR “Taiji” OR “Tai-ji” ) AND AB ( “breast cancer” OR “breast carcinoma” OR “breast neoplasm” OR “breast tumor” ) AND TX “random” |
| Strategy for CNKI:  (SU = '太极' + '太极拳') AND (SU = '乳腺癌' + '乳癌' + ‘乳岩’ + '乳腺肿瘤') AND FT = '随机' |
| Strategy for Wan Fang Database:  主题:(太极 OR 太极拳) and 主题:(乳腺癌 OR 乳癌 OR 乳岩 OR 乳腺肿瘤) and 全部:(随机) |
| Strategy for Sinomed:  ( "太极拳"[常用字段:智能] OR "太极"[常用字段:智能]) AND( "乳腺癌"[常用字段:智能] OR "乳癌"[常用字段:智能] OR "乳岩"[常用字段:智能] OR "乳腺肿瘤"[常用字段:智能]) AND "随机"[全部字段:智能] |
| Strategy for VIP:  (M=太极 OR 太极拳) AND (M=乳腺癌 OR 乳癌 OR 乳岩 OR 乳腺肿瘤) AND U=随机 |

Table 3. The GRADE rubric

| Data source | GRADE domain | Rating |
| --- | --- | --- |
|  | Study design |  |
| n/a | All estimates of effect are meta-analyses of RCTs | 0 |
|  | 1. Risk of Bias * |  |
| (12) | ≥75% RCTs with low Risk of Bias (RoB) for random sequence generation (Y/N) |  |
| (15) | ≥75% RCTs with low RoB for blinding of outcome assessment (Y/N) |  |
| (16) | ≥75% RCTs with low RoB for incomplete outcome data (Y/N) |  |
| (18) | Overall assessment of risk of bias (choose 1)  Low RoB: ≥75% RCTs low RoB in all three categories, or if no information (NI) about categories ≥75% RCTs overall rating of low RoB  Moderate RoB: ≥75% RCTs low RoB in one or two categories, if NI about categories ≥75% RCTs overall rating of moderate RoB  High RoB: <75% RCTs low RoB in all three categories, or if NI about categories <75% RCTs overall rating of low or moderate RoB |  |
| r. s. | Information about sensitivity/subgroup analysis for RoB |  |
|  | a. Low RoB | 0 |
|  | b. Moderate RoB, effect estimate stable with sensitivity/subgroup analysis with only Low RoB RCTs included | 0 |
|  | b. Moderate RoB, no sensitivity/subgroup analysis or effect estimate unstable/becomes non-significant | -1 |
|  | c. High RoB, effect estimate stable with sensitivity/subgroup analysis when high RoB RCTs excluded or only low RoB RCTs included | -1 |
|  | c. High RoB, no sensitivity/subgroup analysis or effect estimate unstable | -2 |
|  | 2. Inconsistency |  |
| k. | Heterogeneity I2 : |  |
| d. e. | Total no. RCTs, and no. in favour of TCC: |  |
| t. u.  & visual | Information about sensitivity/subgroup analysis reduced heterogeneity and direction of effect estimate stable/remains significant, or  effect estimate remains stable with ‘one-out’ study sensitivity analyses,  NOTE: subgroup analysis may not be reliable or indicated if <10 RCTs and ‘one-out’ study sensitivity analysis if <6 RCTs. |  |
|  | Only one RCT in the meta-analysis | 0 |
|  | Heterogeneity I2 ≤75% * | 0 |
|  | Heterogeneity I2 76-89% | -1 |
| & visual | Heterogeneity I2 >90% & all RCTs favour one direction and CIs mostly overlap, & subgroup/sensitivity analysis is indicated and reduces I2 ≤75% and effect estimate stable/ remains significant | -1 |
| & visual | Heterogeneity I2 >90% & mixed direction of results +/- appreciable nonoverlap in CIs (confirm with visual inspection of Forest plot) or NI | -2 |
|  | 3. Imprecision |  |
| e. f. | Sample size: |  |
| g. | Effect estimate: |  |
| h. | 95% CI: |  |
| m. | Minimal important difference (MID): |  |
| n. | Number needed to treat (NNT): |  |
| o. | Absolute Risk/Relative Risk Reduction (RRR): |  |
|  | Optimal information size (OIS): |  |
|  | TSA-IS (Trial Sequential Analysis information size) reached i.e. at least one of the boundaries for benefit, harm or futility are crossed (Y/N): |  |
| external | Minimal clinically important difference (MCID) for MD |  |
| Option 1  OIS | For continuous outcomes:  OIS is met if Meta-analysis (MA) sample size ≥200* or TSA-IS reached |  |
|  | - MA sample size ≥200 & 95%CI does not overlap zero | 0 |
|  | - MA sample size ≥200 & 95%CI overlaps zero & important benefit and harm excluded (95%CI range within ±0.5 SMD, or ±MCID) | 0 |
|  | - MA sample size ≥200 & 95%CI overlaps zero & important benefit or harm included (95%CI either >0.5 or <-0.5 SMD, >MCID or <-MCID) | -1 |
|  | - MA sample size <200 | -1 |
|  | - MA sample size <200 & 95%CI overlaps zero & both important benefit and harm included (95%CI wider than ±0.5 SMD, ±MCID). If NI about CI or MCID, only downrate -1 unless very wide 95%CI. | -2 |
|  | For Relative risk outcomes (OR, RR, HR):  OIS is met if MA sample size >4,000 & no. events >100, or TSA-IS reached.  OIS is not met if MA total no events: <100  Otherwise, calculate OIS: α = 0.05; β = 0.02; 25% RRR (i.e. set RR of intervention at 25% of control group RR) <http://powerandsamplesize.com/Calculators/>  or use Fig 4 and Fig 5 in GRADE guidelines 6. to estimate OIS |  |
|  | - OIS is met & 95%CI does not overlap 1.0 | 0 |
|  | - OIS is met & 95%CI overlaps 1.0 & important benefit and harm excluded (OR, RR of HR 95% CI range between ≥0.75 and ≤1.25) | 0 |
|  | - OIS is met & 95%CI overlaps 1.0 & important benefit or harm included (OR, RR of HR 95% CI <0.75 or >1.25) | -1 |
|  | - OIS is not met | -1 |
|  | - OIS is not met & 95%CI overlaps 1.0 & both important benefit and harm included (OR, RR of HR 95% CI range <0.75 and >1.25) | -2 |
|  | For Absolute risk outcomes (ARR i.e. risk difference):  OIS is met if MA sample size >4,000 & no. events >100, or TSA-IS reached.  OIS is not met if MA total no events: <100  Otherwise, use 25% in Fig 5 in GRADE guidelines 6. to estimate OIS |  |
|  | - OIS is met & 95%CI does not overlap zero | 0 |
|  | - OIS is met & 95%CI overlaps zero & important benefit and harm excluded (for treatment outcomes and non-serious AEs, 95% CI range within ±0.1 , serious AEs ±0.01) | 0 |
|  | - OIS is met & 95%CI overlaps zero & important benefit included and harm excluded (for treatment outcomes and non-serious AEs only, 95% CI >0.1 or <-0.1, for any or serious AEs >0.01 or <-0.01) | -1 |
|  | - OIS is not met | -1 |
|  | - OIS is not met & 95%CI overlaps zero & both important benefit and harm included (for treatment outcomes and non-serious AEs, 95% CI range wider than ±0.1, serious AEs ±0.01) | -2 |
|  | Publication (small study) bias |  |
| c. | < 10 RCTs: n/a | 0 |
| v. w. | > 10 RCTs: PB assessed as not detected or unsure | 0 |
| c. v. w. | > 10 RCTs: PB assessed as “strongly suspected” | -1 |
| c. v. w. | > 10 RCTs: PB not assessed, but “strongly suspected” for another outcome | -1 |
| c. v. w. | > 10 RCTs: PB not assessed, < half studies have sample size <100 | 0 |
| c. v. w. | > 10 RCTs: PB not assessed, > half studies have sample size <100 | -1 |
|  | Other considerations |  |
|  | n/a. Dose response and large effect size only applies to high quality observational studies. Do not rate up for RCT evidence. | 0 |

Table 4. Characteristic of excluded studies

| IDs of Excluded studies | Reasons for excluding |
| --- | --- |
| Galantino ML 2003[1] | The estimates of effect sizes were not available to allow quantitative analysis. |
| Mustian KM 2004[2] | This report did not provide clear information on effect size estimates (HRQoL), which has been included and provided in other reports from the same study. |
| Mustian KM 2006[3] | The outcomes is out of the scoping of this systematic review. |
| Rausch SM 2007[4] | TCC-based exercise duration is only 8 weeks (not more than 12 weeks). |
| Peppone LJ 2010[5] | The outcomes is out of the scoping of this systematic review. |
| Wang YL 2010[6] | The full text does not mention "random" and we do not consider this an RCT. |
| Campo RA 2013[7] | The study included multiple cancers in women, not just breast cancer. |
| Fong SSM 2013[8] | It is a cross-sectional study. |
| Galantino ML 2013[9] | It is a case series study. |
| Robin JLW 2013[10] | The sample sizes of each group were not available to allow quantitative analysis. |
| Irwin MR 2014[11] | The estimates of effect sizes were not available to allow quantitative analysis. |
| Soltero EG 2022[12] | The outcomes is out of the scoping of this systematic review. |
| Zhang JY 2022[13] | TCC-based exercise duration is only 8 weeks (not more than 12 weeks). |

Reference:

[1] Galantino ML, Capito L, Kane RJ, Ottey N, Switzer S, Packel L. The effects of Tai Chi and walking on fatigue and body mass index in women living with breast cancer: a pilot study. Rehabilitation Oncology. 2003;21(1):17-22.

[2] Mustian KM, Katula JA, Gill DL, Roscoe JA, Lang D, Murphy K. Tai Chi Chuan, health-related quality of life and self-esteem: a randomized trial with breast cancer survivors. Support Care Cancer. 2004 Dec;12(12):871-6. doi: 10.1007/s00520-004-0682-6. Epub 2004 Sep 30. PMID: 15599776.

[3] Mustian KM, Katula JA, Zhao H. A pilot study to assess the influence of tai chi chuan on functional capacity among breast cancer survivors. J Support Oncol. 2006 Mar;4(3):139-45. PMID: 16553140.

[4]Rausch SM. Evaluating the Psychosocial Effects of Two Interventions, Tai Chi and Spiritual Growth Groups, in Women with Breast Cancer. Richmond: Virginia Commonwealth University. 2007.

[5] Peppone LJ, Mustian KM, Janelsins MC, Palesh OG, Rosier RN, Piazza KM, Purnell JQ, Darling TV, Morrow GR. Effects of a structured weight-bearing exercise program on bone metabolism among breast cancer survivors: a feasibility trial. Clin Breast Cancer. 2010 Jun;10(3):224-9. doi: 10.3816/CBC.2010.n.030. PMID: 20497921; PMCID: PMC3071508.

[6] Wang YL, Sun XY, Wang YB, Zhou LH, Fang HX, Liu LN. Effects of Taijiquan exercise on limb function and quality of life in patients with breast cancer after surgery. China Sport Science and Technology. 2010;46(5):125-8. doi: 10.16470/j.csst.2010.05.024.

[7] Campo RA, O'Connor K, Light KC, Nakamura Y, Lipschitz DL, LaStayo PC, Pappas L, Boucher K, Irwin MR, Agarwal N, Kinney AY. Feasibility and acceptability of a Tai Chi Chih randomized controlled trial in senior female cancer survivors. Integr Cancer Ther. 2013 Nov;12(6):464-74. doi: 10.1177/1534735413485418. Epub 2013 Apr 25. PMID: 23620504; PMCID: PMC3831606.

[8] Fong SS, Ng SS, Luk WS, Chung JW, Chung LM, Tsang WW, Chow LP. Shoulder Mobility, Muscular Strength, and Quality of Life in Breast Cancer Survivors with and without Tai Chi Qigong Training. Evid Based Complement Alternat Med. 2013;2013:787169. doi: 10.1155/2013/787169. Epub 2013 Apr 23. PMID: 23710237; PMCID: PMC3655570.

[9] Galantino ML, Callens ML, Cardena GJ, Piela NL, Mao JJ. Tai chi for well-being of breast cancer survivors with aromatase inhibitor-associated arthralgias: a feasibility study. Altern Ther Health Med. 2013 Nov-Dec;19(6):38-44. PMID: 24254037.

[10] Robins JL, McCain NL, Elswick RK Jr, Walter JM, Gray DP, Tuck I. Psychoneuroimmunology-Based Stress Management during Adjuvant Chemotherapy for Early Breast Cancer. Evid Based Complement Alternat Med. 2013;2013:372908. doi: 10.1155/2013/372908. Epub 2013 May 14. PMID: 23762127; PMCID: PMC3666296.

[11] Irwin MR, Olmstead R, Breen EC, Witarama T, Carrillo C, Sadeghi N, Arevalo JM, Ma J, Nicassio P, Ganz PA, Bower JE, Cole S. Tai chi, cellular inflammation, and transcriptome dynamics in breast cancer survivors with insomnia: a randomized controlled trial. J Natl Cancer Inst Monogr. 2014 Nov;2014(50):295-301. doi: 10.1093/jncimonographs/lgu028. PMID: 25749595; PMCID: PMC4411534.

[12] Soltero EG, Larkey LK, Kim WS, Rosales Chavez JB, Lee RE. Latin dance and Qigong/Tai Chi effects on physical activity and body composition in breast cancer survivors: A pilot study. Complement Ther Clin Pract. 2022 May;47:101554. doi: 10.1016/j.ctcp.2022.101554. Epub 2022 Feb 15. PMID: 35257993.

[13] Zhang JY, Li SS, Meng LN, Zhou YQ. Effectiveness of a nurse-led Mindfulness-based Tai Chi Chuan (MTCC) program on Posttraumatic Growth and perceived stress and anxiety of breast cancer survivors. Eur J Psychotraumatol. 2022 Feb 3;13(1):2023314. doi: 10.1080/20008198.2021.2023314. PMID: 35140880; PMCID: PMC8820790.

Table 5. Characteristics of the included trials

| **Study ID**^†^**, country** | **Sample size, mean age (years)** | | **AJCC stage** | **Time since anti-cancer treatment completion** | **Intervention type** | **Intervention regimen** | **Control** | **Outcomes** | **Measured time points** |
| --- | --- | --- | --- | --- | --- | --- | --- | --- | --- |
|  | **TCC** | **Control** |  |  |  |  |  |  |  |
| Mustian KM 2008, USA [1-3] | 11, 54.33 | 10, 52.70 | 0–IIIb | 1 week to 30 months | the first 15 moves of the traditional 104-move Yang-style long form | Meet 3 times a week for 60 minute-sessions (10 min of warm-up stretching and basic Chi Kung, 40 min of TCC practice and 5 min of exercise cool-down) | Psychosocial support therapy | QoL (SF-36),  QoL (FACIT-F), IL-8, IL-6, IL-2 | baseline and 12^th^ week |
| Wang YL 2012, China [4] | 63, 47.19 | 71, 47.19 | Ⅰ–III | Treatment not completed | Simplified 24 Form Taichi and Routine rehabilitation training | Same rehabilitation training as control, practice Taichi for twice a day and 20 minutes each time from tenth day after surgery | Routine rehabilitation training | Shoulder function (Constant Murley), QoL (WHOQoL-BREF) | tenth day (baseline), 30^th^ day, 90^th^ day and 180^th^ day after surgery |
| Sun XY 2012, China [5] | 41, 39.18 | 35, 39.18 | Ⅰ | Treatment not completed | Simplified 24 Form Taichi, strength training and Routine rehabilitation training | Same strength training and rehabilitation training as control, Practice Taichi for twice a day and 20 minutes each time from tenth day after surgery | Strength training and routine rehabilitation training | Shoulder function (Constant Murley), QoL (WHOQoL-BREF) | tenth day (baseline), 30^th^ day, 90^th^ day and 180^th^ day after surgery |
| Xiao H 2013, China [6] | 33, 70.5 | 33,  70.3 | Ⅰ–III | Treatment not completed | 24 Form Taichi and Routine rehabilitation training | Same rehabilitation training as control, Practice Taichi for twice a day and 20 minutes each time from tenth day after surgery | Aerobics and routine rehabilitation training | Shoulder function (Neer), QoL (FACT-B) | 15^th^ day (baseline), 90^th^ day and 180^th^ day after surgery |
| Li YQ 2013, China [7] | 29, 47.56 | 28, 47.56 | NI | Treatment not completed | Tai Chi Cloud Hands | Same rehabilitation training as control, Practice Taichi for twice a day and 30 minutes each time from seventh day after surgery | routine rehabilitation training | QoL (WHOQoL-BREF) | seventh day (baseline), 30^th^ day, 90^th^ day and 180^th^ day |
| Larkey LK 2015, USA [8, 9] | 42, 57.7 | 45,  59.8 | 0–III | 6 months to 5 years | A set of 10 movements drawn from the Tai Chi Easy and the “Vitality Method” series of Qigong exercises | Meet twice a week for the first two weeks and then once a week for 60 minute-sessions (a brief time at the beginning of class to socialize with other participants and 45-50 minutes of practice.), practice at home at least 30 minutes a day, 5 days per week at home or in pairs/groups with other classmates | Sham Qigong (A similar set of movements without the focus on breathing and Meditation Movement) | QoL (SF-36), Cognitive function (FACT-COG and WAIS-III), Fatigue (FSI), Depression (BDI), Sleep quality (PSQI) | baseline, 12^th^ week and 24^th^ week of follow-up |
| Lv F 2015, China [10] | 50, 48.61 | 49, 48.61 | NI | Treatment not completed | Simplified 24 Form Taichi and routine rehabilitation training | Same rehabilitation training as control, practice for at least 60 minutes, and at least three times a week | routine rehabilitation training | Shoulder function (Constant Murley), QoL (SF-36) | tenth day (baseline), 30^th^ day and 90^th^ day after surgery |
| Wang HY 2015, China [11] | 75, 48.61 | 74, 48.61 | NI | Treatment not completed | Chen style Taichi and routine rehabilitation training | Same rehabilitation training as control, Practice Taichi for twice a day and 20 minutes each time from tenth day after surgery | routine rehabilitation training | Anxiety (SAS) | tenth day (baseline), 30th day, 90th day and 180th day after surgery |
| Thongteratham N 2015, Thailand [12] | 15,  NI | 15,  NI | 0–IIIb | At least one year | 18-form Taichi Qigong | Meet three times a week for 60 minute-sessions (warm-up for five minutes, exercise for 45 to 50 minutes and cool-down for five to ten minutes), practice for 30 minutes at home, three times per week | Practice as advised in usual care | QoL (FACT-B), Fatigue (FSI) | baseline, 6^th^ week and 12^th^ week |
| Wang HY 2016, China [13] | 44, 53.64 | 46, 51.74 | NI | Treatment not completed | Taichi and routine rehabilitation training | Same rehabilitation training as control, Practice Taichi for twice a day and 20 minutes each time from tenth day after surgery | routine rehabilitation training | Shoulder function (Neer), QoL (FACT-B) | tenth day (baseline), 30^th^ day and 90^th^ day after surgery |
| Zhu JY 2016, China [14, 15] | 47,  NI | 38,  NI | Ⅰ–III | Treatment not completed | Simplified 24 Form Taichi, strength training and Routine rehabilitation training | Same strength training and rehabilitation training as control, Practice Taichi for twice a day and 20 minutes each time from tenth day after surgery | Strength training and routine rehabilitation training | Shoulder function (Constant Murley) | tenth day (baseline), 30^th^ day and 90^th^ day after surgery |
| Irwin MR 2017, USA [16] | 38, 59.6 | 42,  60.0 | NI | At least six months | 20-form Taichi | Meet weekly for 120 minute-sessions (each session provided objectives and learning activities), practice at home | Cognitive behavioral therapy for insomnia | Sleep quality (PSQI) | baseline, 2^nd^ month, 3^rd^ month, 6^th^ month of follow-up and 15^th^ month of follow-up |
| Wang YY 2017, China [17] | 45, 50.5 | 41,  50.5 | Ⅰ–III | Treatment not completed | 24 Form Taichi and routine rehabilitation training | Same rehabilitation training as control, Practice Taichi for twice a day and 20 minutes each time from tenth day after surgery | Strength training and routine rehabilitation training | Fatigue (CFS), QoL (WHOQoL-BREF), sleep quality (PSQI), anxiety (SAS), depression (SDS) | tenth day (baseline), 30^th^ day, 90^th^ day and 180^th^ day after surgery |
| Han Q 2019, China [18] | 23, 46.39 | 21, 45.52 | Ⅰ–III | Treatment not completed | Eight form Taichi and usal care | Same usal care as control, Practice Taichi for twice a day, five days a week and 20 minutes (three min of warm-up stretching, 15 min of TCC practice and 2 min of exercise cool-down) each time | Usal care | Fatigue (PFS-R) | baseline, eighth week and 12^th^ week |
| Chen YH 2021, China [19] | 26,  NI | 26,  NI | Ⅰ–III | Less than 3 months | Simplified 24 Form Taichi and usual rehabilitation training | Same rehabilitation training as control, Practice Taichi for three times a week (twice offline and once online) and 60 minutes each time | usual rehabilitation training | Fatigue (CFS) | Baseline, 16^th^ week |

^†^ The study ID consists of the first author's surname, the capital initials of the first author's first name, and the year the first report of the RCTs was published or submitted as dissertation.

AJCC: American Joint Committee on Cancer; BDI: Beck Depression Inventory; CFS: Cancer Fatigue Scale; Constant Murley: Constant Murley Shoulder Outcome Score; FACT-B: Functional Assessment of Cancer Therapy-Breast; FACT-COG: Functional Assessment of Cancer Therapy-Cognitive Function; FACIT-F: The Functional Assessment of Chronic Illness Therapy-Fatigue; FSI: Fatigue Symptom Inventory; IDS-C: Inventory of Depressive Symptoms; IFN- γ: Interferon-γ; IL-2, 6, 8: Interleukin-2, 6, 8; NI: No Information; PFS-R: the revised piper fatigue scale; PSQI: Pittsburgh Sleep Quality Index; QoL: Quality of Life; SAS: Self-Rating Anxiety Scale; SDS: Self-Rating Depression Scale;; SF-36: the 36-item short-form Medical Outcome Survey; TCC: Tai Chi Chuan; WAIS-III: Wechsler Adult Intelligence Scale-Third Edition; WHOQOL-BREF: World Health Organization Quality of Life Brief Questionnaire.

[1] Mustian KM, Palesh OG, Flecksteiner SA. Tai Chi Chuan for breast cancer survivors. Med Sport Sci. 2008;52:209-217. doi: 10.1159/000134301. PMID: 18487900; PMCID: PMC3927648.

[2] Janelsins MC, Davis PG, Wideman L, Katula JA, Sprod LK, Peppone LJ, Palesh OG, Heckler CE, Williams JP, Morrow GR, Mustian KM. Effects of Tai Chi Chuan on insulin and cytokine levels in a randomized controlled pilot study on breast cancer survivors. Clin Breast Cancer. 2011 Jun;11(3):161-70. doi: 10.1016/j.clbc.2011.03.013. Epub 2011 Apr 20. PMID: 21665136; PMCID: PMC3156577.

[3] Sprod LK, Janelsins MC, Palesh OG, Carroll JK, Heckler CE, Peppone LJ, Mohile SG, Morrow GR, Mustian KM. Health-related quality of life and biomarkers in breast cancer survivors participating in tai chi chuan. J Cancer Surviv. 2012 Jun;6(2):146-54. doi: 10.1007/s11764-011-0205-7. Epub 2011 Dec 10. PMID: 22160628; PMCID: PMC3664952.

[4] Wang YL, Sun XY, Wang YB, Niu F, Liu Y, Zhou LH, Fang HX, Liu LN. Effects of different exercise patterns on upper limb function and quality of life in postoperative patients with breast cancer (不同运动方式对乳腺癌术后患者上肢功能及生活质量的影响). Chin J Phy Med Rehabil. 2012;34(1):64–6. [in Chinese].

[5] Sun XY, Tang LL, Zhou LH, Liu LN, Zhou YP, Wang YB, Wang YL. Influence of comprehensive rehabilitation exercise on upper limb functional rehabilitation and quality of life after breast reconstruction in stage I breast cancer (综合康复锻炼对乳腺癌Ⅰ期乳房重建术后上肢功能康复和生活质量的影响). Chin J Phy Med Rehabil. 2012;34(4):302-5. [in Chinese]

[6] Xiao H, Feng T, Duan YL, Pan JQ. Effects of different rehabilitation exercises on postoperative quality of life and upper limb function in elderly patients with breast cancer (不同康复锻炼法对老年乳腺癌患者术后生活质量及上肢功能的影响). Chin J Gerontol. 2013;33(22):5535-7. [in Chinese].

[7] Li YQ, Li LL, Wei W. Influence of Tai Chi Chuan cloud hand on functional rehabilitation of affected limbs in patients after breast cancer surgery (太极云手对乳腺癌术后患者患肢功能康复的影响). Fujian J Tradit Chin Med. 2013 Oct;44(5):57-8. [in Chinese].

[8] Larkey LK, Roe DJ, Weihs KL, Jahnke R, Lopez AM, Rogers CE, Oh B, Guillen-Rodriguez J. Randomized controlled trial of Qigong/Tai Chi Easy on cancer-related fatigue in breast cancer survivors. Ann Behav Med. 2015 Apr;49(2):165-76. doi: 10.1007/s12160-014-9645-4. PMID: 25124456; PMCID: PMC4329282.

[9] Larkey LK, Roe DJ, Smith L, Millstine D. Exploratory outcome assessment of Qigong/Tai Chi Easy on breast cancer survivors. Complement Ther Med. 2016 Dec;29:196-203. doi: 10.1016/j.ctim.2016.10.006. Epub 2016 Oct 13. PMID: 27912947; PMCID: PMC5326628.

[10] Lv F, Yu Y, Liang D, Li ZM, You W, Zhang B. Effects of Baduanjin Exercise and Shadowboxing on Quality of Postoperation Life for Breast Cancer Patients (八段锦及太极拳锻炼对乳腺癌患者术后生活质量的影响). J Wuhan Inst Phys Educ. 2015 July;49(7):80-3. [in Chinese].

[11] Wang HY. Effects of Taichi exercise pattern on anxiety among postoperative breast cancer patients (太极拳锻炼模式对乳腺癌患者术后焦虑的影响). Chin J Mod Nurs. 2015 Oct;21(28):3386–8. [in Chinese].

[12] Thongteratham N, Pongthavornkamol K, Olson K, Ratanawichitrasin A, Nityasuddhi D, Wattanakitkrilert D. Effectiveness of Tai Chi qi qong program for thai women with breast cancer: a randomized control trial. Pacific Rim Int J Nurs Res. 2015;19(4):280–94.

[13] Wang HY, Dai SJ, Hu M, Yu H, Liu SQ. Effects of Tai Chi exercise on shoulder joint function and quality of life in patients with breast cancer after surgery (太极拳运动对乳腺癌术后患者肩关节功能和生命质量的影响). J Mew Med. 2016;26(3):231-3. [in Chinese].

[14] Zhu JY. The effect of rehabilitation on limb function of patients with breast cancer and analysis of 24 Taijiquan (24式太极拳对乳腺癌患者术后患肢功能的康复效果与分析). Wuhu: Anhui Normal University. 2016. [in Chinese].

[15] Sun XY, Peng YY, Zhu JY, Zhang C, Wang YB, Wang YL. Effects of Taijiquan exercise on functional recovery of patients with breast cancer after surgery and its mechanism (太极拳锻炼对乳腺癌术后患肢功能恢复的影响及机制探讨). Chin J Phys Med Rehabil. 2020 Dec;42(12):1088-90. [in Chinese].

[16] Irwin MR, Olmstead R, Carrillo C, Sadeghi N, Nicassio P, Ganz PA, Bower JE. Tai Chi Chih Compared With Cognitive Behavioral Therapy for the Treatment of Insomnia in Survivors of Breast Cancer: A Randomized, Partially Blinded, Noninferiority Trial. J Clin Oncol. 2017 Aug 10;35(23):2656-2665. doi: 10.1200/JCO.2016.71.0285. Epub 2017 May 10. Erratum in: J Clin Oncol. 2017 Dec 20;35(36):4096. PMID: 28489508; PMCID: PMC5549450.

[17] Wang YY. Effect of Tai Chi Exercise on Cancer-related Fatigue and Quality of Life in Elderly Postoperative Patients with Breast Cancer (太极拳运动对中老年乳腺癌术后患者癌因性疲乏和生活质量的影响). Wuhu: Anhui Normal University. 2017. [in Chinese].

[18] Han Q, Yang L, Huang SY, Zheng MH, Huang SM, Xue H. Study on the effect of eight-style taijiquan on cancer-related fatigue in breast cancer patients (八式太极拳对乳腺癌患者癌因性疲乏影响的研究). J Guangxi Univ Chin Med. 2019 Aug;22(4):30-4. [in Chinese].

[19] Chen YH. Effects of Tai Chi Exercise on Cancer-related Fatigue, Body Composition and Blood Lipid of Breast Cancer Patients after Chemotherapy (太极拳锻炼对乳腺癌患者化疗后癌因性疲乏、身体成分和血脂的影响研究). Wuhan: Wuhan Sports University. 2021. [in Chinese].
